# Hyperspectral imaging in neurosurgery: a review of systems, computational methods, and clinical applications

**DOI:** 10.1117/1.JBO.30.2.023512

**Published:** 2024-11-13

**Authors:** Alankar Kotwal, Vishwanath Saragadam, Joshua D. Bernstock, Alfredo Sandoval, Ashok Veeraraghavan, Pablo A. Valdés

**Affiliations:** a University of Texas Medical Branch, Department of Neurosurgery, Galveston, Texas, United States; b Rice University, Department of Electrical and Computer Engineering, Houston, Texas, United States; c University of California Riverside, Department of Electrical and Computer Engineering, Riverside, California, United States; d Brigham and Women’s Hospital, Harvard Medical School, Department of Neurosurgery, Boston, Massachusetts, United States; e Massachusetts Institute of Technology, David H. Koch Institute for Integrative Cancer Research, Cambridge, Massachusetts, United States

**Keywords:** hyperspectral imaging, fluorescence-guided surgery, neurosurgery, brain tumors

## Abstract

**Significance:**

Accurate identification between pathologic (e.g., tumors) and healthy brain tissue is a critical need in neurosurgery. However, conventional surgical adjuncts have significant limitations toward achieving this goal (e.g., image guidance based on pre-operative imaging becomes inaccurate up to 3 cm as surgery proceeds). Hyperspectral imaging (HSI) has emerged as a potential powerful surgical adjunct to enable surgeons to accurately distinguish pathologic from normal tissues.

**Aim:**

We review HSI techniques in neurosurgery; categorize, explain, and summarize their technical and clinical details; and present some promising directions for future work.

**Approach:**

We performed a literature search on HSI methods in neurosurgery focusing on their hardware and implementation details; classification, estimation, and band selection methods; publicly available labeled and unlabeled data; image processing and augmented reality visualization systems; and clinical study conclusions.

**Results:**

We present a detailed review of HSI results in neurosurgery with a discussion of over 25 imaging systems, 45 clinical studies, and 60 computational methods. We first provide a short overview of HSI and the main branches of neurosurgery. Then, we describe in detail the imaging systems, computational methods, and clinical results for HSI using reflectance or fluorescence. Clinical implementations of HSI yield promising results in estimating perfusion and mapping brain function, classifying tumors and healthy tissues (e.g., in fluorescence-guided tumor surgery, detecting infiltrating margins not visible with conventional systems), and detecting epileptogenic regions. Finally, we discuss the advantages and disadvantages of HSI approaches and interesting research directions as a means to encourage future development.

**Conclusions:**

We describe a number of HSI applications across every major branch of neurosurgery. We believe these results demonstrate the potential of HSI as a powerful neurosurgical adjunct as more work continues to enable rapid acquisition with smaller footprints, greater spectral and spatial resolutions, and improved detection.

## 1 Introduction

Optical imaging approaches have transformed surgery via improved intraoperative detection of both normal and diseased tissues.^1^^–^^5^ Technologies that jointly leverage optics, computational methods, and visualization tools have facilitated this unparalleled transformation, with several successful commercial technologies in areas such as surgical robotics^6^^–^^8^ and image-^2^^,^^3^^,^^5^ and fluorescence-guided^9^^,^^10^ surgery. Image-guided surgery allows for the clinical deployment of optical imaging systems that are non-invasive and non-ionizing, which in turn can be used for intraoperative computer vision,^11^ tactile sensing,^12^ manipulation, and tracking algorithms^13^ that have a relatively compact footprint and allow for rapid acquisition.

As an example, images acquired via a surgical endoscope, processed through computer vision pipelines,^14^ have been used for post-surgical analysis of the surgical workflow,^15^^,^^16^ including recognizing surgical goals, predicting the current task being performed, segmenting and recognizing relevant landmarks during surgery, evaluating the difficulty of the surgical plan, and surgeon skill.^11^ In addition, visual instrument detection and tracking methods for minimally invasive surgeries have been developed and validated on surgical videos.^13^ Autonomous, high-precision, and dexterous surgical instrument manipulation for surgery and remote telesurgery has been made possible^6^^,^^17^^–^^19^ through deep learning methods at precisions previously thought impractical.^8^ Recently developed image-guided surface sensing systems, such as the GelSight sensor,^20^ can provide joint micron-scale topography (2.5-dimensional depth data) and tactile feedback more sensitive than human skin.^21^ The demonstrated effectiveness of these approaches suggests exciting potential prospects for intraoperative applications.

A promising approach in image-guided surgery is hyperspectral imaging (HSI),^22^^–^^25^ which captures wide-field, spectrally resolved images of the surgical field. HSI systems have been deployed successfully for applications in remote sensing, astronomy, agriculture, and surveillance.^26^^–^^28^ Hyperspectral data can be interpreted as an “optical fingerprint” of the material being analyzed (e.g., diffuse reflectance properties) and can be used for material recognition and classification.^29^^–^^32^ Therefore, HSI can enhance visualization of tissue structure and composition in image-guided surgery, aiding in guiding diagnosis and treatment.

In this paper, we review the applications of HSI in neurosurgery, focusing on specific HSI techniques and their medical implementations and benefits in clinical practice. Specifically, we provide the reader with an up-to-date review of how HSI has been implemented clinically and, thus, focus on HSI systems and techniques used in clinical studies only. We begin with preliminaries (Sec. 2), which include an overview of the major subspecialities in neurosurgery (Sec. 2.1), followed by a short review of current HSI techniques (Sec. 2.2). We then discuss the benefits and challenges of HSI in neurosurgery (Secs. 2.3 and 2.4). Next, we proceed with an in-depth review of HSI technologies and their clinical applications for imaging under white light in reflectance mode (Sec. 3) and for imaging fluorescence in fluorescence-guided surgery (Sec. 4). We have broken up Secs. 3 and 4 into technological subsections—imaging hardware and software (Secs. 3.1 and 4.1), datasets (Sec. 3.2), and visualization tools (Sec. 3.3)—and followed them up with clinical implementations of and results from these HSI technologies (Secs. 3.4 and 4.2). By separating each section into technological and clinical subsections, the readers will be able to refer to more detailed technological aspects of HSI (e.g., imaging systems, computational methods, datasets, and visualization techniques) or the clinical results and implementations of these technologies in the various subspecialties of neurosurgery. We also provide in-depth tables that summarize the technological and clinical subsections for ease of reference. Finally, we discuss future perspectives on HSI as a novel tool with the potential to become a standard adjunct in image-guided neurosurgery (Sec. 5).

## 2 Preliminaries

### 2.1 Neurosurgery

Neurosurgery is the branch of medicine that treats disorders of the central nervous system (CNS) or peripheral nervous system (PNS) by physical manipulation, modification, or modulation of anatomical (e.g., the subthalamic nucleus for deep brain stimulation) and pathological (e.g., aneurysm clipping and resection of brain tumors) structures.^33^^–^^35^ In terms of research and clinical techniques, neurosurgery is among the most rapidly developing subspecialties of medicine,^36^ propelled by the interdisciplinary integration of tools from imaging, molecular biology, cancer neuroscience, electrophysiology, brain mapping, neuroengineering, computational biology, bioinformatics, and robotics. Clinically, neurosurgery is composed of the following subspecialties:

“Neurosurgical oncology” is the surgical branch of neuro-oncology focused on the diagnosis, treatment, and long-term management of tumors of the CNS and PNS. Surgical resection is the primary course of treatment for a large set of tumors. The success of tumor resection is one of the most important initial predictors of overall survival and quality of life.^37^^,^^38^ Therefore, the goal of tumor surgery is to maximize the extent of tumor resection (EOR) while preserving the functional brain to ensure high post-operative functional outcomes (i.e., achieving an oncofunctional balance^39^^–^^44^). However, rates of EOR can be as low as 30% as reported by post-operative,^45^ standard-of-care magnetic resonance imaging (MRI) using conventional surgical techniques.

Conventional resections are performed under white light illumination with or without magnification (e.g., using microscopes or surgical loupes). In these procedures, the surgeon uses the cues from visual white light and tactile feedback to determine which tissue to resect and which to preserve.^41^ However, because brain tumors often appear visually similar to normal brain tissue, residual tumors often remain unresected, leading to low rates of maximal EOR. This is especially problematic in infiltrative areas of the most aggressive malignant tumors, such as glioblastomas (GBMs).^41^ Surgical adjuncts such as intraoperative MRI (iMRI), intraoperative ultrasound (US), and neuronavigation can improve visualization and intraoperative surgical decision-making. Despite their benefits, these tools have limitations including disruption of the surgical workflow, inaccurate spatial information due to brain shift, low contrast (normal tissue versus pathology), and high costs.^46^ Therefore, there is an acute need for real-time, high-resolution technologies that accurately delineate tumors from normal brain tissue in neurosurgical oncology.^47^^–^^52^

“Vascular neurosurgery” is the branch of neurosurgery focused on the diagnosis and surgical treatment of blood vessel pathologies of the nervous system.^33^ This encompasses a variety of conditions including aneurysms, arteriovenous malformations (AVMs), stroke, and hemorrhage. The primary aims of surgical treatments include restoring normal blood flow to the brain, preventing blood clot formation and stroke, repairing vascular pathologies (e.g., aneurysms and fistulas), and resecting vascular lesions (e.g., AVMs and cavernomas). Given that the spatial scale of vascular structures in the nervous system is of the order of millimeters, submillimeter precision and real-time intraoperative feedback are critical to safely treat pathologies while preserving normal vasculature. Although intraoperative three-dimensional (3D) digital subtraction angiography provides visualization of the neurovasculature in 3D as well as differentiates its venous and arterial components,^53^ it does not provide direct intraoperative visualization of vasculature and pathology at the tissue level. Intraoperative Doppler US can detect blood flow,^54^ but it is constrained in resolution (i.e., millimeters) and field of view (i.e., single point detection) and is sensitive to patient motion. Intraoperative indocyanine green (ICG) fluorescence angiography provides real-time intraoperative feedback with surface visualization of vasculature using ICG fluorescence, which accumulates in the blood vessels.^55^ However, visualization of vasculature and pathologies is transient (i.e., ICG signal washes out shortly after administration), is useful only for surface imaging, and is not specific to pathologies as it accumulates in all normal and abnormal vasculature.^56^ Therefore, there is an acute need for real-time, non-transient, and highly specific intraoperative imaging technologies that can distinguish between normal and pathological neurovasculature for visual feedback in vascular neurosurgery.

“Functional neurosurgery” is the surgical branch of neurosurgery that treats various chronic neurologic disorders of the brain through functional modification. These disorders include epilepsy, movement disorders, pain, spasticity, and psychiatric illnesses.^33^ One example of functional neurosurgery is the treatment of intractable epilepsy via surgically resecting the epileptogenic area, which is the area of the brain where seizures are believed to originate. The goal of this surgery is to eliminate or decrease the frequency and severity of seizures.^57^ In epilepsy surgery it is important to map out the affected area of the brain, typically with intraoperative electrocorticography (ECoG).^58^ During this procedure, a grid of electrodes is placed on the cortex to measure electrical activity and identify regions with abnormal signals that might indicate seizure origin. However, intraoperative ECoG interrupts the surgical workflow by requiring electrode placement, signal measurement, signal interpretation, electrode removal, and co-registration of electrode locations with signal origins on the brain. In addition, recordings can take a few minutes to complete and interpret. The resolution of ECoG is dependent on the intrinsic spacing within the electrode array, with spatial resolutions of up to a centimeter using conventional grids. There is also a risk of infection associated with the use of such an electrode array with long-term monitoring. As such, imaging techniques that provide visualization of the epileptogenic regions would enable real-time feedback and ideally more accurate identification of the seizure-causing regions. Overall, there is a need for imaging technologies that provide functional neurosurgeons with real-time and highly specific identification of normal and abnormal functions in the nervous system.

“Spine surgery” is the surgical branch of neurosurgery that treats disorders affecting the spinal cord.^33^ Spine surgery can address issues such as spinal deformity, nerve compression, pain, and neurological deficits due to disorders of the spinal cord and nerves. Surgical navigation has become critical in spine surgery to perform accurate manipulation of bony structures while preventing damage to the spinal cord and its surrounding neural elements. Such navigation is typically done with fiducial markers placed on the skin and spine, but these can get obscured, deformed, or displaced during surgery,^59^ compromising accurate real-time guidance. It is therefore clear that to enhance the accuracy and safety of spine surgery, there is a pressing need for non-invasive real-time tracking systems and algorithms. These advanced technologies will provide better guidance during surgical procedures, ensuring more effective treatment of spinal disorders and improved patient outcomes.

“Other subspecialties” of neurosurgery include trauma and peripheral nerve surgery. However, there has been no clinical work with HSI in these subspecialties, so we will not discuss them here.

### 2.2 Hyperspectral Imaging

**Fig. 1:**
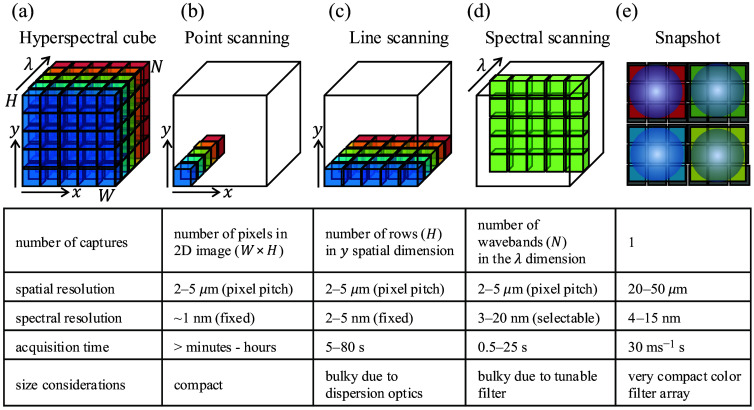
Hyperspectral imaging technologies used in neurosurgery. (a) Hyperspectral image cube is an array of size W×H×N, where W and H are the width and height, respectively, of images in the cube along the x and y spatial dimensions, and N is the number of wavelength channels along the λ dimension. Each W×H channel in the cube is equivalent to an image that would be captured by placing an appropriate bandpass spectral filter in front of the camera. (b) Point scanning methods acquire a complete spectrum at a single (x,y) pixel coordinate (i.e., “point”), scanning along the x and y spatial dimensions to reconstruct the full 3D hyperspectral cube. (c) Line scanning methods acquire 2D data of size W×N along one x spatial dimension, scanning along the y spatial dimension (i.e., “line”) to reconstruct the full 3D hyperspectral cube. (d) Spectral scanning methods acquire 2D data images of size W×H at one λ wavelength channel, scanning along the λ wavelength dimension (i.e., “spectral”) to reconstruct the full 3D hyperspectral cube. (e) Snapshot methods acquire the full 3D hyperspectral image cube of size W×H  ×N with each single acquisition (i.e., “snapshot”).

HSI is the acquisition of high-resolution spectra over a wide field of view. HSI allows for capturing a 3D hyperspectral cube of size H×W×N, where H and W are the height and width of images in the cube, respectively, and N is the number of wavelength channels [Fig. 1(a)]. The value of N roughly distinguishes it from multispectral imaging, a spectrally resolved imaging paradigm that uses fewer, broader spectral bins. Here, we define a multispectral system to have less than 10 wavelength channels (N<10) and a hyperspectral system to have more than 10 (N>10). Each H×W channel in the cube is equivalent to a two-dimensional (2D) image that would be captured by placing an appropriate bandpass spectral filter in front of the camera. Capturing spectral data in addition to spatial information can be used to determine the composition of the contents of the imaged scene.^31^^,^^32^^,^^60^ An in-depth review of the construction and properties of such systems can be found in the literature,^31^^,^^32^ and we discuss only the essentials here. HSI technologies relevant to neurosurgery and their general specifications are illustrated in Fig. 1. Acquisition of a 3D hyperspectral image cube with a 2D camera sensor, however, is not straightforward. Thus, several techniques for the capture of hyperspectral image cubes have been developed, each with its own unique advantages and pitfalls.^61^^,^^62^

“Point scanning methods” (also referred to as whiskbroom scanners) operate using a single detector or a small array of detectors to sequentially scan the scene, capturing spectral data pixel by pixel. Although this method provides high spectral resolution, the point-scanning approach needs M=HW acquisitions, which for megapixel-sized images is time-consuming and limits their use to imaging static scenes and/or small fields of view [Fig. 1(b)].

“Line scanning methods” (also referred to as pushbroom scanners) encode spectral data in one spatial dimension, allowing parallel measurement of the other spatial dimension. Typically, these methods use a linear array of detectors aligned perpendicular to the scanning direction (say, along the H dimension), capturing spectral data row by row. This approach reduces the number of acquisitions to M=W, which significantly reduces acquisition time compared with point scanners. However, the acquisition of thousands of line scans still comes at a high time cost. These are the most widely available systems^63^^–^^66^ used abundantly in HSI applications [Fig. 1(c)].

“Spectral scanning methods” image one spectral channel (i.e., one waveband) in the hyperspectral cube at a time and employ a tunable bandpass spectral filter to capture sequentially 2D images at each spectral channel. Spectral scanners offer the flexibility to acquire cubes over a programmable set of wavelengths with selectable spectral resolution. High-spectral-resolution cubes come at a high time cost, especially when considering their use in the dynamic, fast-paced surgical setting. Typical tunable filters used are liquid crystal tunable filters (LCTFs)^67^ and acousto-optic tunable filters^68^ [Fig. 1(d)].

“Snapshot methods”^69^^–^^71^ capture a hyperspectral cube with complete spatial and spectral information in a single exposure. Snapshot acquisition is achieved by space division multiplexing of the sensor over the spatial and spectral dimensions, similar to a plenoptic camera.^72^ In this approach, the sensor area is distributed over a number of parts equal to the number of spectral channels. Each of these parts images a wide-field image corresponding to one spectral channel, and these parts are stacked together to form the hyperspectral cube. This technology is facilitated by new optical designs incorporating lenslet arrays^70^^,^^71^^,^^73^^,^^74^ and varying filtering and dispersion strategies. This rapid acquisition enables the use of snapshot systems in applications requiring real-time hyperspectral feedback, such as in intraoperative image guidance, where long scan times or bulky scanning hardware can interfere with the surgical workflow. However, space division multiplexing requires a trade-off between spatial and spectral resolutions for equivalent acquisition times—as we increase the number of parts, the sensor is segmented into fewer pixels available for each part [Fig. 1(e)].

“Snapscan systems” combine the benefits of snapshot and line scanning hyperspectral systems. Such systems are built with mosaic filter arrays as in snapshot systems but employ internal scanning of the mosaic and computational reconstructions to yield fast, high-resolution hyperspectral cubes.^75^

“Compressed sensing methods” exploit the regularity in natural signals to obtain an approximation to the hyperspectral cube.^76^ An example of such regularity is the sparsity of individual spectral channels in the spatial frequency domain, which is the subject of a classic signal processing technique called compressed sensing. Such systems have the capability to provide video-rate hyperspectral acquisition with high spatial resolution for scenes that follow its assumptions.^77^ In addition, such methods can also implement programmable spectral filters^78^ in addition to bandpass filters, which allow for matched filtering of spectral signals for classification and segmentation applications.

### 2.3 Benefits of HSI in Neurosurgery

**Fig. 2:**
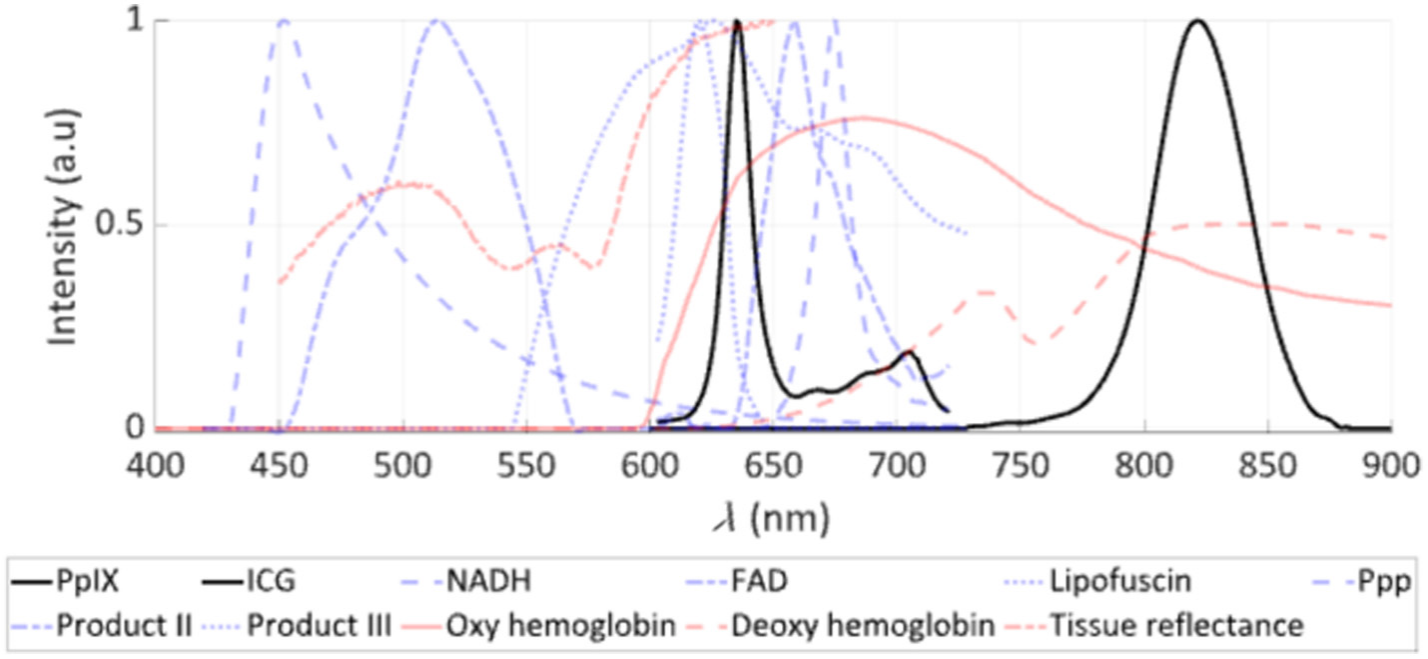
Spectra of fluorophores, chromophores, and reflectance in the visible to near-infrared (NIR) used in HSI for neurosurgery. HSI in neurosurgery has used both exogenous agents, such as 5-aminolevulinic acid that leads to the production of protoporphyrin IX (PpIX), and ICG as key fluorescence biomarkers in fluorescence guided surgery (FGS), with their fluorescence spectra shown in black. Other endogenous fluorophores (e.g., FAD, NADH) are shown in blue, and PpIX photoproducts as well as tissue reflectance and chromophores (e.g., oxy- and deoxyhemoglobin) are shown in red. The y-axis shows the intensity of fluorescence emission, reflectance, or absorption in arbitrary units, and the x-axis shows the wavelength λ, in nanometers.

As mentioned before, the spectrum in one pixel of the hyperspectral cube contains the optical signature or “optical fingerprint” of the imaged scene point at that spatial coordinate (Fig. 2). This fingerprint can include fluorophores [e.g., protoporphyrin IX (PpIX)] and/or chromophores (e.g., oxy- and deoxyhemoglobin) that differentially accumulate in tissues. This fingerprint is representative of the tissue composition of the imaged scene point—typically, bulk brain tissue, arterial blood vessels, venous blood vessels, various types of tumors, and background. HSI is particularly useful when classifying these kinds of tissue because reflectance and fluorescence spectra obtained with the hyperspectral cubes have high discriminative power that has been widely characterized.^79^^–^^83^

As an example of this high discriminative power in the context of vascular neurosurgery, consider a pixel consisting of a blood vessel. The main chromophores involved in the reflectance spectrum of this pixel are oxyhemoglobin and deoxyhemoglobin. The reflectance spectra of deoxyhemoglobin and oxyhemoglobin, which are equal at 545 nm, change rapidly in opposite directions between 545 and 560 nm. Therefore, spectrally resolved imaging in the visible range of the spectrum allows for highly accurate estimates of the relative concentrations of deoxyhemoglobin and oxyhemoglobin, allowing optical measurements of oxygen saturation.

In addition to pixel-wise classification of tissue constituents, hyperspectral data enable other kinds of optical characterization across the surgical field of view. The rich data encoded in each hyperspectral cube offer the potential to extract optical features that would otherwise be impossible to detect visually with the naked eye or with a conventional color image.^67^^,^^84^ For example, spectrally resolved wide-field data have been shown to correct for the distorting effects of tissue optical properties on emitted fluorescence signals,^85^ which opens the possibility for using HSI to evaluate the surgical field of view and provide quantitative, objective measures of fluorescence and therefore absolute fluorophore molar concentrations.^67^

Putting all these capabilities together with modern acquisition techniques from optics and computational imaging, advances in computational methods and hardware, and segmentation and classification with artificial intelligence,^86^ HSI has the potential to be a powerful tool for real-time intraoperative guidance.

### 2.4 Challenges in Current Neurosurgical HSI Approaches

**Fig. 3:**
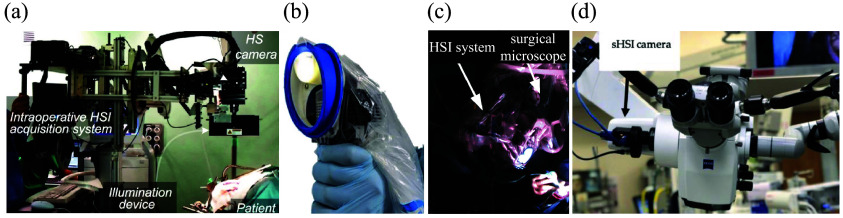
HSI systems in neurosurgery. (a) HELICoiD system uses an exoscope with two line-scan hyperspectral cameras mounted in a confocal configuration. The HELICoiD system fits within a 60×60×90  cm bounding and requires removing the surgical microscope for acquisition, thus interrupting the surgical workflow. (b) Small footprint handheld HSI snapshot system does not require removing the surgical microscope but does not provide the same field of view as seen from the surgeon’s oculars. (c) and (d) HSI systems [spectral scanning in panel (c) and snapscan in panel (d)] mounted on one of the side ports of the surgical microscope enable the acquisition of 3D hyperspectral image cubes co-registered with the surgeon’s field of view with a small physical footprint to seamlessly integrate into the already space-constrained neurosurgical operating room. (a) Adapted from Leon et al.,^88^ under CC-BY 4.0. (b) Adapted from MacCormac et al.,^89^ under CC-BY 4.0. (c) Reproduced from Valdés et al.,^67^ under CC-NC-SA 3.0. (d) Adapted from Kifle et al.,^90^ under CC-BY 4.0.

Translating an optical system for clinical use into the neurosurgical operating room presents unique challenges not encountered in traditional benchtop imaging settings for pre-clinical studies^87^ (Fig. 3). The fundamental principle for translation of a novel HSI system into the operating room is that any system and imaging process must not significantly interfere with or interrupt the neurosurgical workflow; it should enable ease of integration, safety, and efficiency for dynamic intraoperative use. A major practical consideration is the size of the imaging system. The spatial footprint of the optical setup must be as small as possible to seamlessly integrate and “fit” into the already instrument-dense neurosurgical operating room (consisting of, for example, the surgical microscope, US imager, ultrasonic aspirator, neuronavigation, drill, and suction control).

Next, the hyperspectral image captured by the system should be as high-quality as possible, while being as close to real-time as possible (∼10  Hz), consistent with other intraoperative imaging modalities such as US imaging, neuronavigation feedback, microscope visualization, and 3D exoscope imaging. For the hyperspectral data to be useful for surgical guidance, it must fulfill certain basic constraints in addition to real-time acquisition. First, structures in the brain visualized intraoperatively are of the order of millimeters. Therefore, submillimeter resolution over a surgical field of view of the order of centimeters is critical. Second, the spectral bandwidth of the fluorescence peaks of commonly used fluorophores may be as narrow as nanometers, requiring spectral resolutions of a few nanometers. Lastly, as light is split into spectral channels in the already light-starved conditions of fluorescence imaging, the hyperspectral system sensor should have high quantum efficiency, high bit depth, and low dark noise to enable short exposure times.

The speed of hyperspectral acquisition is constrained by the space–spectrum–sensitivity trade-off. Therefore, these conditions are all difficult to satisfy together. The most common, line-scan hyperspectral imagers provide high spectral and spatial resolutions in one spatial dimension [Fig. 3(a)]. However, providing equivalent resolution in the second spatial dimension for surgically relevant scales is time-consuming (typically tens to hundreds of seconds). To be more sensitive to low-intensity fluorescence signals, existing spectral scanning methods [Fig. 3(c)] typically increases exposure times, decreasing hyperspectral cube acquisition rates. Snapshot and snapscan HSI systems^70^^,^^71^^,^^75^^,^^91^ [Figs. 3(b) and 3(d)] can potentially provide fast frame rates for hyperspectral acquisition.^67^^,^^87^^,^^92^^–^^94^ However, they sacrifice spatial resolution to do so, also increasing exposure if increased sensitivity is needed. Managing this balance among the imaging parameters to construct clinically practical and effective systems is one of the most important open problems in neurosurgical HSI.

## 3 Neurosurgical HSI in Reflectance Mode

Traditionally, neurosurgery has been performed under white-light illumination provided by xenon or halogen lamps.^95^ The spectral distributions of such illumination extend across the visible-near-infrared (VIS-NIR) range of the optical spectrum, where the optical properties and reflectance spectra of various types of brain tissue, intracranial structures (e.g., arteries, veins, and nerves), pathologies (e.g., tumors, aneurysms, hemorrhages, and abscess), and their molecular constituents (e.g., oxyhemoglobin and deoxyhemoglobin) have been well-characterized.^79^^–^^83^ Therefore, HSI systems can be used across subspecialties in neurosurgery to serve a common purpose—to determine the composition of what the surgeon sees in the surgical field of view.

**Fig. 4:**
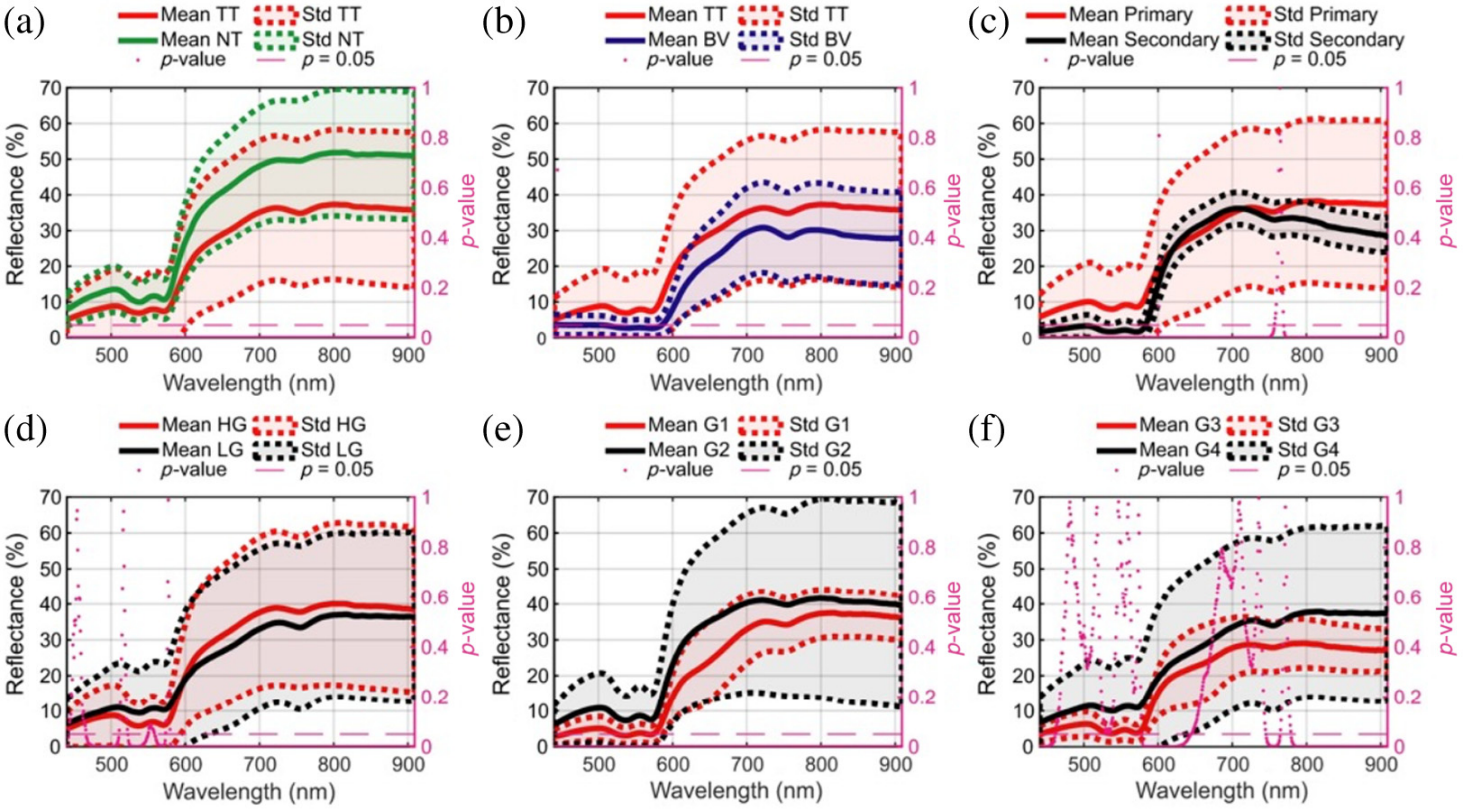
Reflectance spectra of normal brain and brain tumors. (a) and (b) Reflectance spectra of normal tissue (NT) and tumor tissue (TT) and blood vessels (BVs) are significantly different in the visible-NIR regime. (c)–(f) Significant differences are observed in the reflectance spectra from different grades of primary tumors (low grade, high grade, grade 1, grade 2, grade 3, and grade 4) as well as in metastases (i.e., secondary). These differences in reflectance spectra enable the classification of the field of view into the brain parenchyma, blood vessels, and tumor tissue, along with subclassification into arteries, veins, and various tumor types and grades. The y-axis shows the reflectance of tissue in arbitrary units, and the x-axis shows the wavelength λ in nanometers. Adapted from Leon et al.,^88^ under CC-BY 4.0.

For example, in neurosurgical oncology, the aim is to determine the presence or absence of tumor in the field of view, to classify tumor type, and to identify background tissue (Fig. 4). In vascular neurosurgery, the aim is to image blood perfusion and oxygen saturation. In functional neurosurgery, the aim is to identify the epileptiform regions by measuring neurovascular coupling. In spine surgery, the aim is to track surgical field skin features for intraoperative navigation without the use of fiducial markers. Here, we provide a detailed presentation of the optical designs of HSI systems that have been implemented in the neurosurgical operating room. These systems along with their parameters are discussed in Sec. 3.1 and summarized in Table 1.

**Table 1:** Technical specifications of current hyperspectral imaging systems in neurosurgery (as applied in individual work).

HIS system	Clinical application	HIS tech	Sensor tech	Wavelength range	Spectral bands	Spectral resolution	Field of view	Pixel resolution	Spatial resolution	Frame rate per line/channel)	Total time
Neurosurgical oncology—reflectance											
Headwall Hyperspec^®^ VNIR A-series, Headwall Hyperspec^®^ NIR 100/U	Tumor segmentation from normal tissue, blood, and background^112^^–^^115^^,^^146^^,^^147^	Line detection, scanned manually on a translation stage	Silicon CCD, InGaS	400 to 1000 nm 900 to 1700 nm	826 172	2 to 3 nm 5 nm	230 mm (max) × 129 mm 230 mm (max) × 153 mm	1787 (max) × 1004 479 (max) × 320	129 μm 480 μm	90 fps 100 fps	80 s 40 s
Specim ImSpector VNIR V10-E spectrograph	Brain tissue classification^97^	Pushbroom	CCD	400 to 1000 nm	1040	2.8 nm	N/S	N/S	N/S	N/S	N/S
Headwall Hyperspec^®^ VNIR A-series (only)	Tumor segmentation from normal tissue, blood, and background^88^^,^^96^^,^^99^^,^^116^^,^^119^^,^^122^^,^^127^^,^^131^^,^^132^^,^^140^^,^^148^^,^^174^	Line detection, scanned manually on a translation stage	Silicon CCD	400 to 1000 nm	826	2 to 3 nm	230 mm (max) × 129 mm	1787 (max) × 1004	129 μm	90 fps	80 s
IMEC snapshot multispectral SM5x5	Brain tissue classification^234^	Snapshot	CMOS	676 to 954 nm	25	12 nm (inf.)	N/S	410 × 216	N/S	N/A	N/S
Ximea MQ022HG-IM-SM5X5-NIR	Brain tissue classification^98^^,^^150^	Snapshot	CMOS	665 to 975 nm	25	14 nm (inf.)	N/S	409 × 217	N/S	170 fps	70 ms
TIVITA tissue camera	Brain tissue classification^87^	Pushbroom	CMOS	500 to 1000 nm	100	5 nm	60 mm × 70 mm	640 × 480	110 to 125 μm (inf.)	100 fps	∼6 s
IMEC snapscan VNIR 150	Brain tumor identification^93^^,^^141^	Snapscan	CMOS	470 to 900 nm	150	10 to 15 nm	N/S	3600 × 2048	N/S	N/A	2 to 20 s
BaySpec OCITM-D-2000Ultra-compact hyperspectral	Brain tumor identification^90^	Snapshot	N/S	475 to 875 nm	35 to 40	12 to 15 nm	N/S	500 × 270	N/S	50 fps	20 μs−1 s
Cubert Ultris X50	Evaluation of snapshot hyperspectral imaging in neurosurgery^89^	Snapshot	CMOS	350 to 1000 nm	155	4 nm	N/S	570 × 570	N/S	1.5 fps	0.67 s
Neurosurgical oncology—fluorescence											
Custom multispectral system	Residual brain tumor detection^209^	Spectral scanning	CCD	495 to 720 nm	5	20 nm	3 cm diameter	755 × 484	150 μm	N/S	15 s
CRi VariSpec LCTF + PhotonMax	Brain tumor identification^107^	Spectral scanning	CCD	400 to 720 nm	33	20 nm at 550 nm	25.4 mm	512 × 512	200 μm	6 s	120 s
CRi VariSpec LCTF + pco.pixelfly	PpIX concentration estimation^67^^,^^84^	Spectral scanning	CCD	400 to 720 nm	55 (WL) 75 (FL)	5 nm (WL) 3 nm (FL)	10 to 50 mm × 7.5 to 40 mm	696 × 520	N/S	N/S	4 to 16 s
CRi VariSpec LCTF + pco.edge	PpIX concentration estimation^211^^,^^219^	Spectral scanning	CMOS	400 to 720 nm	N/S	N/S	N/S	2560 × 2160	N/S	50 ms	10 to 30 s
CRi VariSpec LCTF + hNü EMCCD	PpIX concentration estimation^217^	Spectral scanning	EMCCD	400 to 720 nm	52 (WL) 52 (FL)	3 nm (WL) 3 nm (FL)	$20\;{\mathrm{cm}}^{2}$	512 × 512	N/S	10 to 100 ms	1.04 to 10.4 s
CRi VariSpec LCTF + ORCA-Flash4.0	PpIX concentration estimation^221^	Spectral scanning	EMCCD	400 to 720 nm	33 (WL) 33 (FL)	10 nm	N/S	1024 × 1024	N/S	>100 ms	26.4 s
CRi VariSpec LCTF + Sony IMX252	PpIX concentration estimation^94^^,^^198^^,^^199^^,^^201^^,^^202^^,^^222^^,^^223^^,^^226^	Spectral scanning	sCMOS	420 to 730 nm	63 (WL) 104 (FL)	5 nm (WL) 3 nm (FL)	N/S	∼2048×∼2048 (variable across work)	N/S	>100 ms	N/S
Senop HSC-2	PpIX concentration visual versus machine threshold comparison^206^	Spectral scanning	CMOS	510 to 635 nm	4	20 nm	N/S	1024 × 1024	N/S	65.9 ms	0.46 s
Vascular neurosurgery											
Eba Japan HSC1700	Oxygenation mapping^101^^,^^156^	Pushbroom	CCD	400 to 800 nm	81	5 nm	N/S	640 × 480	N/S	30 fps	5 to 16 s
IMEC snapshot multispectral	Distinguishing blood and blood vessels^157^	Snapshot	CCD	480 to 630 nm	16	15 nm	$13\;{\mathrm{cm}}^{2}$	256 × 512	100 μm	20 fps	<50 ms
Functional neurosurgery											
CRi VariSpec LCTF + pco.pixelfly	Imaging epileptiform regions^104^	Spectral scanning	CCD	480 to 660 nm	4	N/S	N/S	1392 × 1024	N/S	N/S	N/S
IMEC snapshot multispectral	Imaging neurovascular coupling^92^^,^^160^	Snapshot	CMOS	480 to 630 nm	16	15 nm	$13\;{\mathrm{cm}}^{2}$	256 × 512	100 μm	10 to 20 fps	25 to 95 ms
Ximea MQ022HG-IM-SM5X5-NIR	Intraoperative brain mapping^152^^,^^163^	Snapshot	CMOS	665 to 960 nm	25	13 nm (inf.)	N/S	409 × 217	N/S	170 fps	14 fps
Spine surgery											
Quest Medical Imaging BV Hyperea	Markerless positioning during spine surgery^59^	Snapshot	Silicon CCD	450 to 950 nm	41	∼12 nm	15×15 cm	500 × 250	30 μm (inf.)	16 fps	N/S
IMEC snapscan VNIR, Photonfocus (MV0-D2048x1088-C01-HS02-160-G2)	Tissue classification in spine surgery^167^	Snapscan Snapshot	CCD	470 to 900 nm 665 to 975 nm	150+25	10 to 15 nm 15 nm	N/SN/S	3650 × 2048 409 × 217	N/S N/S	N/S 50 fps	2 to 40 s 1 s

N/S, not specified; N/A, not applicable; WL, white light; FL, fluorescence

To process, interpret, and visualize the hyperspectral data captured with these HSI systems, accompanying computational methods have been developed. For example, in neurosurgical oncology, a number of classification and segmentation algorithms label every pixel in the surgical field as normal tissue, tumor (primary or secondary),^96^ necrosis,^97^ blood vessel (artery or vein),^98^ dura mater,^98^ hypervascularized tissue,^99^ skull,^100^ or background. Similarly, spectral fitting methods process HSI data captured during vascular and functional neurosurgery to yield perfusion and oxygenation maps.^92^^,^^101^^–^^105^ Along with details on optical hardware, we also present a brief review of these computational methods in Sec. 3.1 and summarize their pipelines, validation methods, and best results in Table 2. For a more detailed review of such computational methods, please refer to Massalimova et al.^106^

**Table 2:** Computational methods developed for hyperspectral imaging in neurosurgery.

Objective	Pre-processing	Input format	Target	Algorithm	Validation standard	Validation method	Best validation metrics/results	Speed	Hardware/algorithm
Neurosurgical oncology—reflectance									
Tumor identification, Fabelo et al.^113^	A. Spatial non-uniformity correction B. Dark frame subtraction and flat-fielding C. Denoising D. Spectral normalization	Pixel-wise	Classes: tumor tissue, normal tissue, and background	SVM	Tumor histopathology results from regions of interest	Tenfold cross-validation on mixed-patient pixel spectra	87% overall accuracy 78% tumor sensitivity	N/A	Hyperspec^®^ Data Processing Unit
				MLP			97% overall accuracy 93% tumor sensitivity		
				RF			99% overall accuracy 99% tumor sensitivity		
Tumor identification and type prediction, Fabelo et al.^96^	A. Dark frame subtraction and flat-fielding B. Spatial denoising C. Spectral smoothing and cropping D. Spectral normalization	Pixel-wise	Classes: tumor tissue and normal tissue Subclasses: primary tumor and metastasis	RF	Visual assessment and tumor histopathology results from region of interest	Tenfold cross-validation on mixed-patient pixel spectra	99.7% overall accuracy 99.7% tumor sensitivity 99.6% subclass accuracy 100% subclass sensitivity	N/A	Kalray many-core processor
Tumor identification speedup, Madroñal^97^	A. Cropping of regions of interest B. Automatic specularity and background removal	Pixel-wise	Classes: tumor tissue, normal tissue, and necrosis	SVM	*Ex vivo*	Tenfold cross-validation on mixed-sample pixel spectra	N/S	2.3 Hz	Kalray massively parallel processor array MPPA-256-N
Dimensionality reduction with semantic tumor segmentation, Ravi et al.^114^	A. Dark frame subtraction and flat-fielding B. Spatial denoising C. Spectral smoothing and cropping D. Spectral normalization E. Novel deep learning–based embedding (FR-t-SNE)	Cube	Classes: tumor tissue and normal tissue Subclasses: primary tumor and their types and metastasis and their origin (nine total)	Discrete cosine transform–based semantic texton forest	Visual assessment and tumor histopathology results from region of interest	Sixfold cross-validation	72% overall accuracy 53% tumor sensitivity (due to inter-patient variability) 92% tumor specificity	40 s/cube	Intel Xeon E7-8890 v424 cores
Tumor and blood vessel identification, Fabelo et al.^122^	A. Dark frame subtraction and flat-fielding B. Spatial denoising C. Spectral smoothing and cropping D. Spectral normalization E. FR-t-SNE embedding	Cube	Classes: tumor tissue, normal tissue, blood vessel, and background	Mixed supervised–unsupervised pipeline	Visual assessment and tumor histopathology results from region of interest	Tenfold cross-validation	99% to 100% overall accuracy 98% to 100% tumor sensitivity	1 min/cube	Kalray MPPA-256-N
Tumor and blood vessel identification, tumor type prediction, and speedup, Fabelo et al.^115^	A. Dark frame subtraction and flat-fielding B. Spatial denoising C. Spectral smoothing and cropping D. Spectral normalization	Cube	Classes: tumor tissue, normal tissue, and blood vessel Subclasses: primary tumors, metastasis, and their origin (eight total)	Mixed supervised–unsupervised pipeline	Visual assessment and tumor histopathology results from region of interest	Tenfold cross-validation	98% overall accuracy Unspecified sensitivity	1 min/cube	Kalray MPPA EMB01 board
Brain tissue classification, Fabelo et al.^131^	A. Dark frame subtraction and flat-fielding B. Spatial denoising C. Spectral smoothing and cropping D. Spectral normalization	Cube	Classes: tumor tissue, normal tissue, hypervascularized tissue, and background	Combined 1D DNN and 2D CNN	Visual assessment and tumor histopathology results from region of interest	Leave-one-patient-out cross-validation	80% overall accuracy 42% tumor accuracy	1 min/cube	NVIDIA Quadro K2200 GPU
		Cube		2D deep convolutional neural network			77% overall accuracy 42% tumor accuracy	1 min/cube	
		Pixel-wise		1D deep neural network			77% overall accuracy 40% tumor accuracy	10 s/cube	
Hyperspectral band selection, Martinez et al.^99^	A. Dark frame subtraction and flat-fielding B. Spatial denoising C. Spectral cropping and normalization D. Spectral resampling	Pixel-wise	Classes: tumor tissue, normal tissue, hypervascularized tissue, and background	SVM	Visual assessment and tumor histopathology results from region of interest	Leave-one-patient-out cross-validation	With the top 2.5% most significant spectral bands: 77% overall accuracy 57% tumor sensitivity	N/S	Algorithm
Brain tissue classification, Fabelo et al.^174^	A. Dark frame subtraction and flat-fielding B. Spatial denoising C. Spectral smoothing and cropping D. Spectral normalization	Cube	Classes: tumor tissue, normal tissue, hypervascularized tissue, and background	2D deep convolutional neural network	Visual assessment and tumor histopathology results from region of interest	Leave-one-patient-out cross-validation	85% overall accuracy 41% tumor sensitivity	1 min/cube	NVIDIA Titan-XP GPU
		Pixel-wise		1D deep neural network			84% overall accuracy 42% tumor sensitivity	10 s/cube	NVIDIA Quadro K2200 GPU
Tumor and blood vessel identification, Manni et al.^132^	A. Dark frame subtraction and flat-fielding B. Spectral cropping C. Spectral band selection	Pixel-wise	Classes: tumor tissue, normal tissue, blood vessel, and background	SVM	Visual assessment and tumor histopathology results from region of interest	Leave-one-patient-out cross-validation	76% overall accuracy 43% tumor sensitivity	N/S	N/S
		Cube		2D convolutional neural network			72% overall accuracy 14% tumor sensitivity		NVIDIA Titan-XP GPU
		Cube		2D–3D hybrid convolutional neural network			80% overall accuracy 68% tumor sensitivity		
		Pixel-wise		1D deep neural network			78% overall accuracy 19% tumor sensitivity		
Tumor identification, Martínez-González et al.^119^	A. Dark frame subtraction and flat-fielding B. Spectral smoothing C. Spectral band selection	Pixel-wise	Classes: tumor tissue and normal tissue	Linear scalar SVM	Visual assessment and tumor histopathology results from region of interest	Unspecified data split	89% overall sensitivity	<1 s	Intel Core i5
Gray–white matter classification, Lai et al.^234^	Dark frame subtraction and flat-fielding	Pixel-wise	Classes: gray matter and white matter	SVM	Visual assessment and tumor histopathology results from region of interest	Leave-one-patient-out cross-validation	96% overall sensitivity 89% overall specificity	N/S	N/S
Brain tissue classification, Cruz-Guerrero et al.^116^	Dark frame subtraction and flat-fielding	Pixel-wise	Classes: tumor tissue, normal tissue, hypervascularized tissue, and background	Blind linear unmixing with end-member estimation (EBEAE)^144^^,^^235^	Visual assessment and tumor histopathology results from region of interest	Leave-one-patient-out cross-validation	67% to 76% overall accuracy 30% to 50% tumor sensitivity	29 to 32 s/cube	Algorithm
Tumor and blood vessel identification and tumor type prediction, Ruiz et al.^98^	A. Dark frame subtraction and flat-fieldingB. Spectral correction and normalization	Pixel-wise	Classes: tumor tissue, normal tissue, venous blood vessel, arterial blood vessel, and dura mater	SVM	Visual assessment and tumor histopathology results from region of interest	Leave-one-patient-out cross-validation	75% to 97% overall median accuracy	N/S	N/S
				RF			55% to 97% overall median accuracy		
Hyperspectral cube fusion, Leon et al.^147^	A. Dark frame subtraction and flat-fieldingB. Spatial denoisingC. Spatial upsampling for NIR image	Cube	Fused hyperspectral image	Spatial registration using SURF and MSER detectors via a projective transform	N/A	Structural similarity index (SSIM) among gray reconstructions from transformed cubes	0.78 SSIM 21% accuracy improvement	N/S	N/S
Brain tissue classification, Hao et al.^135^	A. Dark frame subtraction and flat-fielding B. Spatial denoising C. Spectral smoothing and band selection D. Spectral normalization	Cube	Classes: tumor tissue, normal tissue, hypervascularized tissue, and background	CNN	Visual assessment and tumor histopathology results from region of interest	Leave-one-patient-out cross-validation	97% overall accuracy 91% tumor sensitivity	N/S	NVIDIA GeForce RTX 2080Ti GPU
Hyperspectral band selection, Baig et al.^118^	A. Dark frame subtraction and flat-fielding B. Spatial denoising C. Spectral smoothing and downsampling D. Spectral normalization	Pixel-wise	Classes: tumor tissue and normal tissue	Empirical mode decomposition	Visual assessment and tumor histopathology results from region of interest	Leave-one-patient-out cross-validation	88% overall accuracy for the top 2.5% most significant bands	N/S	Algorithm
Brain tissue classification, Urbanos et al.^150^	A. Dark frame subtraction and flat-fielding B. Spectral correction and normalization	Pixel-wise	Classes: tumor tissue, normal tissue, venous blood vessel, arterial blood vessel, and dura mater	SVM	Visual assessment and tumor histopathology results from region of interest	Leave-one-patient-out cross-validation	60% overall accuracy 20% tumor sensitivity	N/S	N/S
		Pixel-wise		RF			53% overall accuracy 11% tumor sensitivity		
		Cube		CNN			49% overall accuracy 32% tumor sensitivity		
Hyperspectral image denoising, Sun et al.^236^	N/S	Cube	Denoised image	TV-regularized denoising	N/A	N/A	N/A	N/S	N/S
Brain tissue classification, Ayaz et al.^136^	A. Dark frame subtraction and flat-fielding B. Spectral dimensionality reduction and sensitivity correction	Cube	Classes: tumor tissue, normal tissue, hypervascularized tissue, and background	3D CNN	Visual assessment and tumor histopathology results from region of interest	80:10:10 data split	>99% overall accuracy 99% tumor sensitivity	N/S	NVIDIA GeForce RTX 5000 GPU
Brain tissue classification, Wang et al.^134^	A. Dark frame subtraction and flat-fielding B. Spectral dimensionality reduction and sensitivity correction	Cube	Classes: tumor tissue, normal tissue, hypervascularized tissue, and background	CNN	Visual assessment and tumor histopathology results from region of interest	500:1 data split	>99% overall accuracy 99% tumor accuracy	N/S	N/S
Brain tissue classification, Cebrián et al.^137^	N/S	Cube	Classes: tumor tissue, normal tissue, blood, and meninges	Deep recurrent neural network	Visual assessment and tumor histopathology results from region of interest	Fivefold cross-validation	>99% overall AUC >99% tumor AUC	N/S	N/S
Brain tissue classification, La Salvia et al.^140^	A. Dark frame subtraction and flat-fieldingB. Spectral band selection	Cube	Classes: tumor tissue, normal tissue, hypervascularized tissue, and background	CNNUNet++, DeepLabV3+ architectures	Visual assessment and tumor histopathology results from region of interest	Leave-one-patient-out cross-validation	76% tumor accuracy 76% tumor sensitivity	0.29 s	NVIDIA GeForce RTX 2080 GPU
Testing deep learning and classical machine learning algorithms for low-grade gliomas, Giannantonio et al.^141^	Spectral band selection	Pixel-wise	Classes: tumor tissue and normal tissue	SVM	Visual assessment	75:25 data split	91% overall accuracy 92% overall sensitivity	N/S	NVIDIA GeForce RTX 3090 GPU
		Pixel-wise		RF			86% overall accuracy 88% overall sensitivity		
		Pixel-wise		MLP			92% overall accuracy 91% overall sensitivity		
		Cube		CNN			81% overall accuracy 80% overall sensitivity		
Hyperspectral band selection, Zhang et al.^145^	A. Dark frame subtraction and flat-fielding B. Spectral normalization	Pixel-wise	Classes: tumor tissue, normal tissue, blood vessel, and background	Data gravitation and weak correlation	Visual assessment and tumor histopathology results from region of interest	Fivefold cross-validation	90% to 98% overall accuracy	1 s	Algorithm
Tumor and blood vessel identification, Leon et al.^88^	A. Dark frame subtraction and flat-fielding B. Spatial denoising C. Spectral cropping, smoothing, and downsampling D. Spectral normalization	Cube	Classes: tumor tissue, normal tissue, blood vessel, and background	Mixed supervised–unsupervised pipeline	Visual assessment and tumor histopathology results from region of interest	60:20:20 data split Fivefold cross-validation	87% overall accuracy 58% tumor accuracy	N/S	N/S
Pediatric tumor identification, Kifle et al.^90^	None	Pixel-wise	Classes: tumor tissue and normal tissue	RF	Visual assessment	70:30 data split	83% to 85% overall accuracy	N/S	N/S
Tumor and blood vessel identification, Sancho et al.^152^	A. Dark frame subtraction and flat-fielding B. Spectral normalization and correction	Cube	Classes: tumor tissue, normal tissue, blood vessel, and dura mater	Mixed supervised–unsupervised pipeline	Visual assessment and tumor histopathology results from region of interest	80:20 data split	95% overall AUC 95% tumor AUC	14 fps	NVIDIA GeForce RTX 3090 GPU
Brain tissue classification, Martín-Pérez et al.^100^	A. Dark frame subtraction and flat-fielding B. Spatial denoising C. Spectral cropping and correction D. Spectral normalization	Pixel-wise	Classes: tumor tissue (with subclasses), normal tissue, arterial and venous blood vessels, dura mater, and skull	RF	Visual assessment and tumor histopathology results from region of interest	80:15:5 data split	57% tumor AUC (with snapshot HSI) 65% tumor AUC (with line scan HSI)	N/S	N/S
HSI-MR registration, Villa et al.^173^	None	Cube	MRI-HSI fusion	Depth-based 3D registration	Actuator position	N/A	∼4 mm registration error	5 s	N/S
Brain tissue classification, Zhang et al.^142^	A. Dark frame subtraction and flat-fielding B. Spectral normalization	Cube	Classes: tumor tissue, normal tissue, blood vessel, and background	CNN	Visual assessment and tumor histopathology results from region of interest	Unspecified data split	97% overall accuracy	90 to 100 s	N/S
Neurosurgical oncology—fluorescence									
PpIX concentration estimation, Valdés et al.^67^ and Valdés et al.^84^	Spectral interpolation	Pixel-wise	PpIX concentrations	Fitting to known fluorophore mixture spectra and empirical correction algorithm	Liquid tissue-mimicking phantoms	Phantom correction accuracy	24% PpIX concentration accuracy 20 ng/ml detection threshold	4 to 8 s	N/A
PpIX concentration estimation, Valdés et al.^211^	Spectral interpolation	Pixel-wise	PpIX concentrations	Empirical correction algorithm	Liquid tissue-mimicking phantoms	Phantom correction accuracy	6% PpIX concentration accuracy 20 ng/ml detection threshold	1 to 2 s	N/A
PpIX concentration estimation, Jermyn et al.^217^	Spectral interpolation	Pixel-wise	PpIX concentrations	Empirical correction algorithm	Liquid tissue-mimicking phantoms	Phantom correction accuracy	Best corrected fluorescence fit $R^{2}=0.931\;\mathrm{ng}/\mathrm{ml}$ detection threshold	N/S	N/A
PpIX concentration estimation, Xie et al.^221^	Dark frame subtraction and flat-fielding	Cube	PpIX concentrations	Spatially regularized reconstruction	Liquid tissue-mimicking phantoms	Phantom correction accuracy	Best corrected fluorescence fit $R^{2}=0.9310\;\mathrm{ng}/\mathrm{ml}$ detection threshold	N/S	N/A
PpIX concentration estimation, Bravo et al.^219^	Dark frame subtraction and flat-fielding	Pixel-wise	PpIX concentrations	Fitting to known fluorophore mixture spectra and empirical correction algorithm	Liquid tissue-mimicking phantoms	Phantom correction accuracy	Ground truth to estimate linear fit $R^{2}=0.9814\;\mathrm{ng}/\mathrm{ml}$ detection threshold	N/S	N/A
Fluorescence component spectra identification, Black et al.^199^	Dark frame subtraction and flat-fielding	Pixel-wise	Significance of auto-fluorescence	Fitting to autofluorescence and PpIX spectra	Fluorescence spectra from biopsies	Spectral unmixing fit quality	In weakly fluorescing areas, 82% lower error for five-component spectral fitting as opposed to PpIX 635 peak only	N/A	N/A
Tumor property classification, Black et al.^222^	A. Dark frame subtraction and flat-fielding B. Spectrally constrained dual-band normalization	Pixel-wise	Tumor type, grade, glioma margins, and IDH mutation prediction	RF and multilayer perceptron	Fluorescence spectra from biopsies	Fivefold cross-validation	87% tumor type accuracy 96% tumor grade accuracy 86% margin accuracy 93% IDH margin accuracy	N/A	N/S
Joint correction and unmixing of fluorescence spectra, Black et al.^226^	N/S	Cube	Corrected fluorescence spectra	1D convolutional neural network in a mixed supervised–unsupervised framework	Liquid tissue-mimicking phantoms/pig brain homogenates	Pearson correlation coefficients between known and predicted concentrations	r=0.997 for phantoms r=0.990 for pig brain homogenates	N/A	N/S
Fluorescence component spectra identification and significance, Black et al.^223^	A. Dark frame subtraction and flat-fielding B. Spectrally constrained dual-band normalization	Pixel-wise	Fluorescence spectrum library	Sparse non-negative Poisson regression	Fluorescence spectra from biopsies, simulated data	Data distribution analysisSpectral component abundances	Data distribution is 82% closer to Poisson than Gaussian in terms of KL divergenceEach library component is present in >7% of the dataset	N/A	N/A
Vascular neurosurgery									
Cerebral oxygenation mapping, Mori et al.^101^	A. Spectral smoothing and cropping B. Spectral normalization	Pixel-wise	Oxygen saturation	Fitting to known hemoglobin and oxyhemoglobin spectra	N/A	N/A	N/A	10 s/cube	N/A
Distinguishing blood and blood vessels, Laurence et al.^157^	A. Dark frame subtraction and flat-fielding B. Denoising C. Spatial registration to account for breathing	Pixel-wise	Oxygen saturation temporal dynamics	Fitting to known hemoglobin and oxyhemoglobin spectra and Fourier transform	Electrocorticography recordings	Visual overlay comparison	N/A	25 s/cube	N/A
Diagnosing cerebral hyperperfusion, Iwaki et al.^156^	ROI selection and outlier rejection	Pixel-wise	Oxygen saturation	Fitting to known hemoglobin and oxyhemoglobin spectra	Visual assessment and co-registered SPECT images	Comparison against SPECT	85% hyperperfusion sensitivity	N/S	N/A
Co-designing hemodynamic and brain mapping, Caredda et al.^165^	N/A	Pixel-wise	Oxygen saturation and cytochrome-c-oxidase concentration	Fitting to known hemoglobin, cytochrome-c-oxidase, and oxyhemoglobin spectra and Monte Carlo light transport simulation	Ground truth from light transport simulation	Comparison against ground truth	Concentration estimation errors: 0.5% oxyhemoglobin 4.4% hemoglobin 15% oxCCO	N/A	N/A
Functional neurosurgery									
Imaging seizures within surgery, Noordmans et al.^104^	A. Dark frame subtraction and flat-fielding B. Spectral normalization	Pixel-wise	Oxygen saturation temporal dynamics	Fitting to known hemoglobin and oxyhemoglobin spectra	Electrocorticography recordings	Visual overlay comparison	N/A	N/S	N/A
Imaging neurovascular coupling, Pichette et al.^92^	A. Dark frame subtraction and flat-fielding B. Spectral filter response linear correction C. Spatial registration to account for breathing D. Spatial cropping to region of interest	Pixel-wise	Oxygen saturation temporal dynamics	Fitting to known hemoglobin and oxyhemoglobin spectra	N/A	N/A	N/A	N/S	N/A
Metabolic brain mapping, Caredda et al.^163^	A. Spatial registration to account for breathing B. Spectral smoothing	Pixel-wise	Oxygen saturation and cytochrome-c-oxidase concentration	Fitting to known hemoglobin and oxyhemoglobin spectra	Electrical brain stimulation data	Visual overlay comparison and normalized cross-correlation coefficient	Correlation coefficients over time range of interest: 0.76 oxyhemoglobin 0.86 hemoglobin 0.84 oxCCO	N/S	Intel Core i5-7200U
Imaging hemodynamic response to interictal epileptiform discharges, Laurence et al.^160^	A. Dark frame subtraction and flat-fielding B. Spatial registration to account for breathing C. Spatial cropping to region of interest D. Outlier rejection	Pixel-wise	Oxygen saturation	Fitting to known hemoglobin and oxyhemoglobin spectra	Electrocorticography recordings	Visual overlay comparison	N/A	N/S	N/A
Spine surgery									
Positioning feedback and navigation, Manni et al.^59^	A. Dark frame subtraction and flat-fielding B. Spatial denoising C. Spectral band selection	SURF/DELF/MSER features^237^^–^^239^	Feature displacement	k-nearest neighbors	Fiducial markers	Comparison between detected and actual marker locations	250 μm marker localization error	N/S	N/A

N/S, not specified; N/A, not applicable; WL, white light; FL, fluorescence; SURF, speeded up robust features; MSER, maximally stable extremal regions; SSIM, structural similarity index measure; KL, Kullback–Leibler; SPECT, single-photon emission computed tomography.

### 3.1 Imaging Hardware and Software

#### 3.1.1 Neurosurgical oncology

HSI for use in neurosurgical oncology was introduced by Gebhart et al.^107^ in 2007 with the use of a Varispec VIS-20 LCTF from Cambridge Research Instruments, Inc.^108^ coupled with a 512×512 PhotonMax electron multiplying charge-coupled device (EMCCD) camera^109^ mounted on a surgical microscope to measure intraoperative autofluorescence and diffuse reflectance spectra with acquisition times of 5 min. Here, the authors did not solely use reflectance but rather both reflectance and autofluorescence measurements to determine a reflectance/autofluorescence ratio for optimal identification of tumor tissue. Similar to the previous approach, Valdés et al.^67^ used a Varispec LCTF coupled with a pco.pixelfly charge-coupled device (CCD) camera^110^ on a surgical microscope (Zeiss OPMI Pentero) [Fig. 3(c)] to measure the reflectance and fluorescence spectra in a fluorescence correction algorithm to enable more accurate measurement of tissue fluorophores during brain tumor resection. Here, both approaches did not solely use reflectance measurements for tissue identification. Instead, they coupled their reflectance measurements with fluorescence to enable tumor tissue identification, which will be discussed in more detail later (see Sec. 4). It was not until 2016 with the kickoff of the European Hyperspectral Imaging Cancer Detection (HELICoiD) project^111^ and the development of the HELICoiD demonstrator by Salvador et al.^112^ and Fabelo et al.,^113^ where HSI of reflectance was used solely for tumor tissue identification.

The HELICoiD demonstrator consists of a pair of line sensor hyperspectral cameras mounted on a custom optical breadboard in the operating room [Fig. 3(a)]. These cameras, bought off-the-shelf from Headwall Photonics,^64^ are the CCD-based Hyperspec^®^ VNIR A-series operating in the VIS-NIR wavelength range (400 to 1000 nm, 826 spectral bands, 2- to 3-nm resolution, 90  frames/s) and the InGaS-based Hyperspec^®^ NIR 100/U operating in the NIR short-wave infrared (SWIR) wavelength range (900 to 1700 nm, 172 spectral bands, 5-nm resolution, 100  frames/s). The cameras are set up in a confocal stereo configuration with matched fields of view, at an imaging distance of 40 cm and surgical field clearance of 29 cm. The entire imaging assembly is mounted on a translation stage to implement pushbroom scanning [Fig. 1(c)]. The demonstrator system uses a 150-W quartz–tungsten–halogen (QTH) bulb with a spectral range of 400 to 2200 nm, passed through an optical fiber to a cold light emitter. This ensures that the heat from the QTH bulb is not transmitted to the tissue to avoid tissue damage. Follow-up work in the HELICoiD project used other hyperspectral line cameras, such as the Specim ImSpector^®^ VNIR V10-E spectrograph^66^ (400 to 1000 nm, 2.8-nm resolution) by Madroñal et al.^97^ and the Headwall Hyperspec^®^ NIR X-Series^63^ (900 to 1700 nm, 166 spectral bands, 100  frames/s) by Ravi et al.^114^ in linear scanning configurations to capture hyperspectral datasets.

In the initial HELICoiD pilot study, several pixel-wise classification algorithms were used on the data collected with the HELICoiD demonstrator to test the potential of reflectance spectra in tumor resection. These include support vector machines (SVMs), multilayer perceptrons (MLPs), and random forests (RFs) implemented on parallel processing platforms such as the Headwall Hyperspec^®^ Data Processing Unit^112^^,^^113^ (31 images from 22 procedures on primary glioblastomas and 135k labeled spectra from the HELICoiD demonstrator) and the Kalray MPPA-256-N HPC device^96^ (13 images from 13 procedures on glioblastomas and metastases and 25k labeled spectra from the HELICoiD demonstrator). The training data consisted of mixed-patient pixel-wise spectra from intraoperative hyperspectral cubes with pathologist-labeled ground truth classification labels. These were tested on data from both HELICoiD cameras separately, and the VIS-NIR data were shown to be most effective with the RF classifier, providing cross-validated accuracy, sensitivity, and specificity greater than 99% for mixed-patient pixel-wise three-class classification.^96^^,^^113^ Subsequently, this classification scheme with a larger dataset (36 cubes from 22 patients, >375  k labeled spectra from the HELICoiD demonstrator) has been integrated into a mixed supervised–unsupervised framework to provide fast intraoperative visualization^115^ with a total per-frame acquisition and processing time of 1 min at an overall accuracy greater than 98% for five-class classification (including blood vessels). Further work has extended and improved these results with techniques such as blind linear unmixing^116^^,^^117^ and empirical mode decomposition,^118^ shown SVMs effective for identifying malignant tumor phenotypes,^119^ and demonstrated estimation of the molecular composition of brain tissues in real time.^120^

Further, to ease the time and computational complexity of working with high-dimensional hyperspectral data (hundreds of wavelength channels across millions of pixels) and improve the semantic consistency of segmentation, dimensionality reduction with manifold embedding has been employed.^114^ This method uses a deep learning–based modified version of the T-distributed stochastic neighbor (t-SNE) manifold embedding algorithm,^121^ called fixed-reference t-SNE (FR-t-SNE). This non-linear embedding method attempts to preserve local spatial regularity (nearby pixels represent the same class with high probability) while still capturing high-level global features (pixel classes). The possibility for generalization of this method was evaluated by testing the model on patient data from a different set of individuals, with around 72% overall accuracy and 53% tumor sensitivity for four-class classification (33 images from 18 patients, captured with the HELICoiD demonstrator). A combination of the above pixel-wise and dimensionality-reduced classifiers to create a joint spatio-spectral classifier has been shown by Fabelo et al.^122^ to have an overall accuracy greater than 99%, with a speed-up of >4.5 to 8.5× achieved with hardware acceleration (five cubes from five patients and 45k labeled spectra from HELICoiD demonstrator).

Various hardware acceleration platforms have been explored to speed up the classification computation by individually optimizing the components of these classifiers. The linear kernel SVM^113^ has been sped up 3 to 5× on massively parallel processor arrays^97^ and system-on-chip architectures^123^^,^^124^ and 90× on graphics processing unit (GPUs);^125^ dimensionality reduction with principal component analysis (PCA) for data preprocessing^115^ has been sped up 36× using multiple central processing unit (CPU) compute cores;^126^ k-nearest neighbor classification^115^^,^^122^^,^^127^ has been sped up 30 to 66× on GPUs; and k-means clustering^115^^,^^122^ has been sped up 150× on GPUs.^128^ Jointly implementing the entire pipeline with PCA on a multi-GPU^129^ platform has resulted in a total speed-up of 180× over the serial platforms, resulting in processing times being reduced from several hundreds of seconds to tens of seconds.^129^ The effect of optimizing the data-type representation of the hyperspectral images and their storage in memory has been explored for lower-throughput processing.^130^

**Fig. 5:**
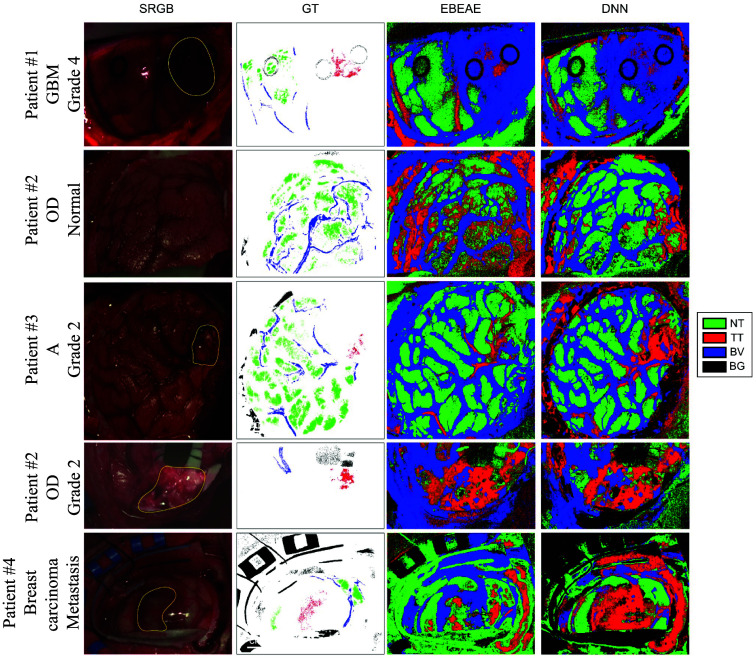
Classifying brain tissue types based on reflectance spectra. Left to right: intraoperative hyperspectral reflectance imaging on four patients with glioma grades 2 and 4 using the HELICoiD system (patient 1 in row 1, patient 2 in rows 2 and 4, patient 3 in row 3, and patient 4 in row 5), white-light synthetic RGB image reconstructed from the hyperspectral cube with tumor regions marked in yellow and biopsy sites with black circles, ground truth–labeled pixels and pixel classifications using linear unmixing methods [extended blind end-member and abundance extraction (EBEAE)],^117^^,^^144^ and a two-layer pixel-wise DNN.^131^ The four classes are NT, TT, BV, and BG. EBEAE yields around 60% overall accuracy, 30% tumor sensitivity, and 85% tumor specificity, whereas the DNN yields 85% overall accuracy, 65% tumor sensitivity, and 95% tumor specificity with fivefold cross-validation on mixed-patient pixel-wise data. GBM, glioblastoma; OD, oligodedrogioma; A, astrocytoma. Adapted from Leon et al.,^88^ under CC-BY 4.0.

Recently, deep learning has been applied to tumor identification in both deep fully connected per-pixel and convolutional spatio-spectral configurations.^131^^,^^132^ This generalizes the hyperspectral data embedding and classification features for the embedded data while allowing for fast computation on the GPU. In combination with unsupervised clustering techniques and minimal user guidance, these accuracies rise to 77% to 78% for one-dimensional (1D) spectral deep neural networks (DNNs),^131^^,^^132^ 72% to 77% for 2D convolutional neural networks (CNNs),^131^^,^^132^ 80% for a combination of 1D DNN and 2D CNN,^131^ and 80% for 3D spatio-spectral CNNs^132^ (with datasets consisting of eight cubes from six patients and 82k labeled spectra;^131^ 12 cubes from 12 patients and 116k spectra,^132^ both from the HELICoiD). Other deep learning architectures^133^^–^^143^ have also produced comparable results with the potential for fast hyperspectral brain structure classification. Figure 5 shows examples of the HELICoiD demonstrator during brain tumor surgery for tissue classification using unmixing methods and deep neural networks.

Manual initial feature engineering has also been attempted to provide better pre-processed data as input for classification algorithms. For example, by selecting the most relevant spectral bands using iterative combinatorial optimization algorithms,^99^ correlation-based ranking,^145^ and deep learning.^141^ In addition, registered pairs of VIS-NIR and NIR images from the HELICoiD demonstrator have been analyzed for spectral similarities between classes to ignore non-distinctive samples.^146^

The two data streams from the visible and near-infrared (VNIR) and NIR cameras in the original HELICoiD setup^112^^,^^113^ need to be fused to create a single hyperspectral cube^146^ to add more useful data to the computational methods described above. Therefore, a new version of the demonstrator has been proposed by Leon et al.,^147^ where the confocal stereo configuration is changed to make the camera axes parallel. This changes the transformation between the two camera viewpoints from a projection to a translation, allowing for less spatial and radiometric distortion of the captured spectra. Combined with spatial and spectral upsampling, hyperspectral cubes are generated at the original spatial resolution and two wavelength ranges (641 spectral bands between 435 and 901 nm and 144 spectral bands between 956 and 1638 nm), resulting in a 21% accuracy increase as compared with using just the VNIR camera on a synthetic material database.

Because the HELICoiD system is mounted on a platform separate from the surgical microscope, it interrupts the surgical workflow due to the need for physical translation of the HELICoiD system prior to data acquisition [Fig. 3(a)]. To prevent such movement, Mühle et al.^87^ designed a workflow with a TIVITA^®^ VIS-NIR tissue hyperspectral camera (500 to 1000 nm, 100 spectral bands, 5-nm spectral resolution, 640×480 output pixels, ∼100  frames/s, ∼6  s/cube)^65^ mounted onto surgical microscope oculars. However, as the cameras used in the above projects can capture only one-dimensional spatial slices, physical scanning of the cameras in one dimension across the surgical field of view is required to capture the entire hyperspectral cube. Thus, this system can capture nanometer-resolution megapixel intraoperative surgical datasets (comparable with previous systems^88^^,^^122^^,^^147^^,^^148^) at the cost of ∼5  s per capture. Data captured from this system yields 99% accuracy and greater than 98% sensitivity for tumor detection (one patient, 29k labeled spectra). However, given the time requirement for data acquisition of a single hyperspectral cube, it has had limited utility for routine clinical use as it significantly interrupts the surgical workflow, which precludes performing the resection under continuous feedback from the HSI system.

Therefore, snapshot HSI systems such as the Ximea Corporation MQ022HG-IM-SM5X5-NIR (665 to 975 nm, 25 spectral bands, 409×217  pixels, 170  frames/s)^149^ based on the IMEC SM5x5 NIR sensor, the Cubert Ultris X50 (350 to 1000 nm, 155 spectral bands, 570×570  pixels, 1.5  frames/s),^91^ the Senop HSC-2 (freely selectable bandwidths and resolutions)^73^ and the BaySpec OCI-2000 Series snapshot hyperspectral imagers (475 to 875 nm, 35 to 40 spectral bands, 50  frames/s)^74^ have been explored as potential alternatives^89^^,^^90^^,^^98^^,^^150^^–^^154^ [Fig. 1(d)]. These can be mounted either by themselves^89^^,^^98^^,^^150^^–^^152^ or coupled to a surgical microscope^90^^,^^93^^,^^153^^,^^154^ to minimize disturbance to the surgical workflow [Fig. 3(d)]. In addition, systems that fuse the advantages of snapshot and line scanning hyperspectral acquisition, called snapscan systems (such as the IMEC Snapscan VNIR,^75^^,^^93^ 470 to 900 nm, 150 spectral bands, 3600×2048  pixels, 2- to 20-s acquisition), coupled with surgical microscopes have been used for intraoperative imaging.^141^ These systems have been used to develop machine learning-based classification (e.g., SVM, decision tree, and RF classifiers^90^^,^^93^^,^^98^^,^^151^) and convolutional neural networks,^153^ with similar results—for instance, a system with the Senop HSC-2 camera reported accuracies around 98%.^153^

#### 3.1.2 Vascular neurosurgery

A major goal in vascular neurosurgery is to restore healthy blood flow to structures in the brain and prevent ischemia (i.e., oxygen-starved), clots, and bleeding. Healthy blood flow leads to an adequate supply of oxyhemoglobin to tissue. Therefore, oxygen saturation (i.e., ratio of oxyhemoglobin to total hemoglobin) in bulk tissue is used as a measure of tissue health and adequate oxygen delivery to tissues.

Hyperspectral oxygen saturation estimation was first used for intraoperative imaging of the cerebral cortex in the superficial temporal artery (STA)–middle cerebral artery (MCA) bypass by Mori et al.^101^ Hyperspectral cubes were acquired with a standalone HSC1700 line scanning camera originally developed for the TAIKI Hyperspectral EO Mission (400 to 800 nm, 81 spectral bands, 640×480  pixels, 5- to 16-s acquisition).^155^ A mixed spectrum consisting of hemoglobin, deoxyhemoglobin, and bulk tissue scattering was fit,^102^ and oxygen saturation was estimated from these proportions. This study found that the STA-MCA anastomosis increased the oxygen saturation distal to the anastomosis corresponding to MCA territory brain regions in two patients with moyamoya disease and two with occlusion of the internal carotid artery. Further, Iwaki et al.^156^ also found that HSI could detect cerebral hyperperfusion following this anastomosis in five patients with moyamoya disease. These results showcased the potential of hyperspectral data in vascular neurosurgery for hemodynamic imaging (i.e., imaging of blood flow and tissue perfusion).

Fu et al.^103^ developed an LCTF-based HSI system coupled with a Zeiss surgical microscope tested to predict cerebral ischemia in rats. Unlike the prior work which fit spectra to estimate oxygen saturation, the authors used an empirical measure to estimate oxygen saturation and tissue perfusion. This work showed that the ratio of tissue reflectance around 545 nm to reflectance around 560 nm could identify early brain ischemia in a rat stroke model. Their method works using the reflectance of deoxyhemoglobin and oxyhemoglobin, which are equal at 545 nm but change rapidly in opposite directions between 545 and 560 nm, yielding a high predictive power for estimating low oxygen saturation.

Further, a snapshot hyperspectral system from IMEC with filters mosaiced on a CCD sensor (480 to 630 nm, 16 spectral bands, 256×512  pixels, 20  frames/s) was used by Laurence et al.^157^ to distinguish between blood vessels and bleeding in the cortex in three patients. Diffuse reflectance spectra measured by the camera are fit to a model consisting of a combination of oxyhemoglobin, deoxyhemoglobin, and tissue absorption.^102^ The estimated oxyhemoglobin proportion is Fourier-transformed to calculate its temporal frequency distribution. It was inferred that the healthy regions where the oxygen saturation is driven by the respiratory rate (cortex and blood vessels) had a first harmonic temporal frequency of around 0.23 Hz, with a significant second harmonic at 0.46 Hz. Meanwhile, bleeding varied more significantly than the heart rate at a frequency of around 1.3 Hz, which allowed for accurate identification of the vessels. Noordmans et al.^158^ used intraoperative HSI and found that these slow, sinusoidal hemodynamic oscillations displayed a stable and reproducible frequency in four epilepsy patients, which included non-lesional, focal cortical dysplasia and dysembryoplastic neuroepithelial tumor, emphasizing the possibility to generalize this method.

#### 3.1.3 Functional neurosurgery

Epilepsy surgery requires the mapping of metabolically active brain regions, including epileptogenic regions, that demand more oxygen and blood. This link between neuronal activity and changes in blood flow and oxygenation is commonly referred to as neurovascular coupling.^159^ As seizures result from intense, uncontrolled neuronal activity, regions of the brain exhibiting seizure activity are highly metabolically active and as such display differences in their neurovascular coupling compared with regions not exhibiting seizure activity.

**Fig. 6:**
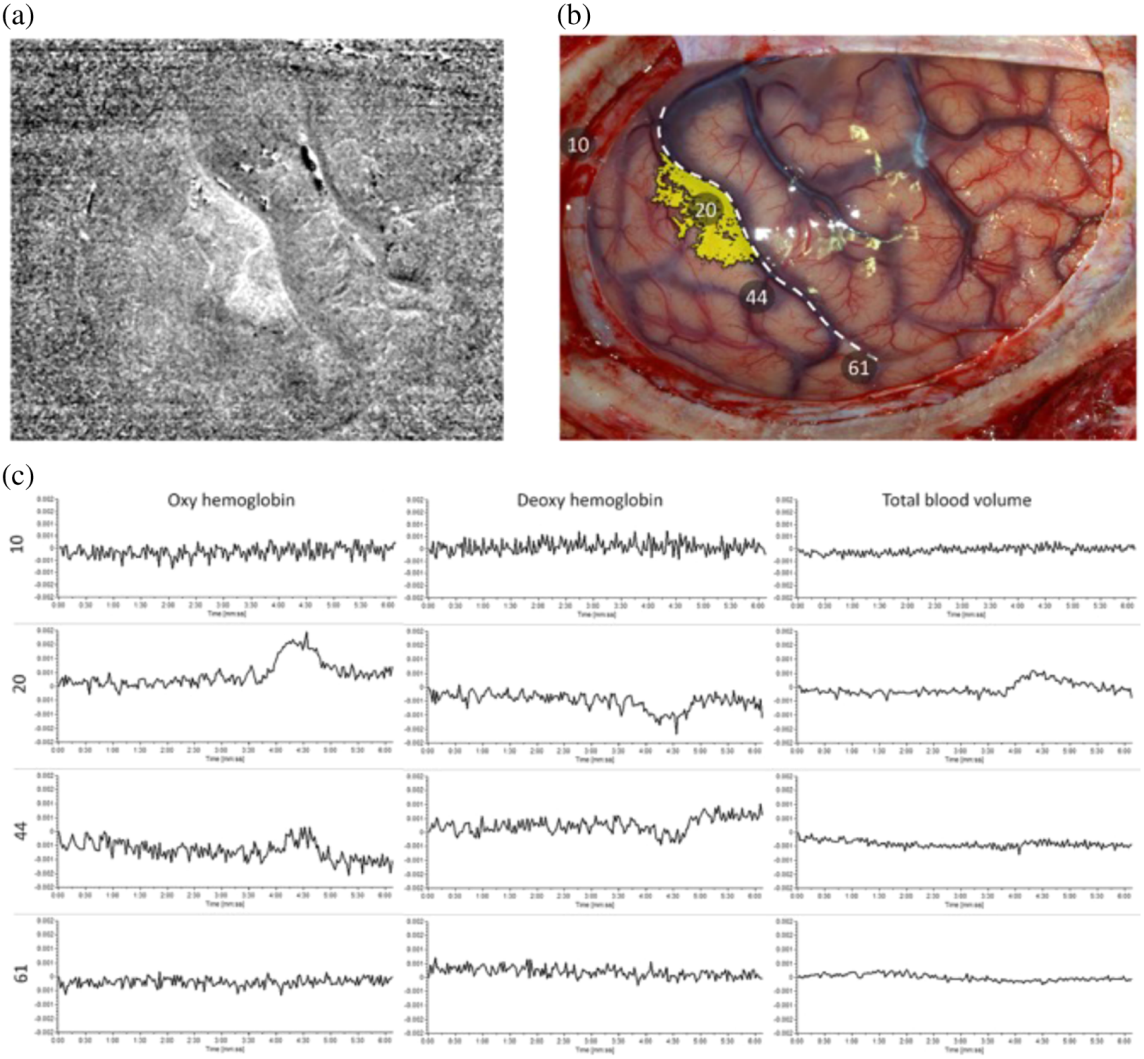
HSI to map seizures intraoperatively. (a) Local increase in oxygenation during seizure: oxygenation changes estimated from oxyhemoglobin concentration during a seizure. (b) Area matched to a photo of the cortex: overlay of oxygenation changes on an RGB image of the brain cortex, which correlates with electrical recordings of seizure activity measured via electrocorticography. Position 20 corresponds to the sensory cortex of the hand where positive seizure activity was recorded and HSI measured higher oxygenation. Reproduced from Noordmans et al.,^104^ with permission from John Wiley & Sons, Inc. (c) Relative concentration as a function of time.

The first use of HSI for evaluating neurovascular coupling dynamics in epilepsy intraoperatively was in 2013 by Noordmans et al.,^104^ where one patient with intractable sensorimotor seizures of the left hand was imaged using an LCTF-based system (Varispec VIS^108^ filter with a pco.pixelfly camera,^110^ 1392×1024  pixels) coupled to a Zeiss Pentero surgical microscope (Fig. 6). In this work, the entire cerebral cortex was imaged over the span of 7 min, and the area of increased oxyhemoglobin at the start of seizure activity matched the epileptogenic zone. Subsequently, Laurence et al.^105^ further validated this finding in 12 epilepsy patients, which included non-lesional, focal cortical dysplasia type and heterotopia. The authors found that regions of seizure activity were isolated with an intraoperative HSI system.

Further, a snapshot hyperspectral system from IMEC with filters mosaiced on a CCD sensor (480 to 630 nm, 16 spectral bands, 256×512  pixels, 10 to 20  frames/s) coupled with a Zeiss Pentero microscope was used for intraoperative hemodynamic imaging on one patient undergoing epilepsy surgery resection by Pichette et al.^92^ at video rates. Laurence at al.^160^ tested this system to measure the interictal discharges in eight patients with non-lesional or subcortical heterotopias undergoing epilepsy surgery, where unsupervised clustering of oxygenation correlated well with direct electrical measurements of the imaged cortex.

Lastly, HSI has been used for intraoperative optical functional brain mapping with a three-chromophore [oxyhemoglobin, deoxyhemoglobin, and oxygenated cytochrome-c-oxidase (oxCCO)^161^^,^^162^] system by Caredda et al.^163^ Incorporating oxCCO into the model introduces a direct measure of cellular metabolism. This work used a Ximea Corporation MQ022HG-IM-SM5X5-NIR hyperspectral camera (665 to 960 nm, 25 spectral bands, 409×217  pixels, 14  frames/s)^149^ to measure the tissue reflectance spectra while the patient was repetitively clenching his fist. These reflectance spectra were fit to the model, and the resulting concentration maps were thresholded to identify areas of high oxygenation and metabolism, which were found to strongly correlate with those identified with gold standard direct electric brain stimulation. In addition to incorporating oxCCO, Caredda et al.^164^ have demonstrated blind unmixing using non-negative matrix factorization to account for two metabolic biomarkers strongly correlating with direct electrical brain stimulation on 12 patients undergoing resection for a brain tumor near the motor cortex.

HSI techniques in vascular and functional neurosurgery have both used oxygen saturation and hemodynamics. Therefore, optimal schemes for measuring the two simultaneously have been studied in Caredda et al.^165^ with Monte Carlo simulations of hemodynamic signals following neuronal firings. These schemes select specific combinations of NIR spectral bands from the hyperspectral image to ensure minimal errors in estimating the proportions of oxyhemoglobin, deoxyhemoglobin, and oxCCO, therefore seeking to achieve accurate metabolic and hemodynamic inferences. Simulations for the specific system designed and implemented in previous work^166^ augmented with a Ximea MQ022HG-IM-SM5X5-NIR hyperspectral camera^149^ were performed considering the effect of realistic factors such as spectral cross-talk and Gaussian noise on the estimation error. This study found that 21 to 22 spectral bands were enough to compute tissue chromophore proportions accurately (0.5% error for oxyhemoglobin, 4.4% error for deoxyhemoglobin, and 15% error for oxCCO), whereas 10 to 12 spectral bands provided a similar performance. The general approach implemented with this Monte Carlo simulation can potentially be used outside hemodynamic imaging in neurosurgical oncology and spine surgery to determine the optimal spectral signatures for tissue identification tasks using HSI.

#### 3.1.4 Spine surgery

HSI has been hypothesized to be useful in spine surgery as another form of surgical navigation to enable surgeons to operate without causing injury to surrounding neural elements. To demonstrate the utility of HSI in non-invasive patient positioning and navigation, a Hyperea snapshot hyperspectral camera from Quest Medical Imaging BV (450 to 950 nm, 41 spectral bands, 500×250  pixels, 16  frames/s) has been used to track skin features pre-operatively by Manni et al.^59^ Based on hyperspectral data collected from 17 healthy volunteers with breathing-based motion, submillimeter feature tracking was demonstrated using both handcrafted features and deep learning.

The first demonstration of intraoperative HSI in spine surgery was on a single patient undergoing spinal fusion by Ebner et al.^167^ This work showed the utility of both a stand-alone snapscan system from IMEC (470 to 900 nm, 150 spectral bands, 3650×2048  pixels, 2 to 40 s acquisition)^75^ and a stand-alone Photonfocus MV0-D2048x1088-C01-HS02-160-G2 NIR snapshot camera (665 to 975 nm, 25 spectral bands, 409×217  pixels, 50  frames/s)^168^ separately. These systems were used to capture video-rate hyperspectral reflectance data for tissue types and implant materials encountered in spinal surgery (skin, fat, muscle, bone, connective tissue, dura, and screws) in a bovine calf cadaver. The experience of the surgical team using this system intraoperatively was that it integrated smoothly into the surgical workflow.

### 3.2 Datasets

The HSI systems described in Sec. 3.1 have produced rich datasets of intraoperative hyperspectral data. Some of this data are available in the public domain for use by researchers who do not have access to or do not have the resources for constructing and deploying their own HSI systems. We describe publicly available datasets, including those captured for individual projects.

#### 3.2.1 Neurosurgical oncology

The HELICoiD project has produced the following datasets available^169^ by contacting the authors.

• HELICoiD Sample In-Vivo HS Human Brain Database: This dataset from Fabelo et al.^122^ contains five VIS-NIR hyperspectral cubes of grade IV glioblastoma multiforme (GBMs) taken during procedures on five different adult patients with a Hyperspec^®^ VIS-NIR A-series camera. These acquisitions took place at the University Hospital Doctor Negrin of Las Palmas de Gran Canaria (Spain) and the University Hospital of Southampton (United Kingdom). These cubes are 1004×1010 in spatial dimension and contain 826 spectral bands between 400 and 1000 nm. A subset of 44,555 marked pixels from these images with types identified with high confidence by the operating neurosurgeon has been labeled in one of four categories: normal tissue, tumor tissue, blood vessel, and background with a biopsy smear of their corresponding tissue. To reduce human error, this entire gold standard labeling process was done in a computer-assisted manner with a custom-built graphical unit interface and a programmable angle threshold from known tissue-type spectra with the spectral angle mapper algorithm.^170^ This data can be downloaded from the authors’ webpage.^169^
• HELICoiD Full In-Vivo HS Human Brain Database: This extended version of the previous dataset from Fabelo et al.^148^ contains 36 hyperspectral cubes from 22 patients with the same VIS-NIR camera, cropped to the region of interest (ROI). It contains data not only on GBMs but also on grade II and III oligodendrogliomas, meningiomas, and metastases from renal, lung, and breast carcinomas. The gold standard labeling was done in the same semi-automatic way as in the previous database. The password for this repository can be obtained by contacting the authors.^169^
• HELICoiD Enhanced In-Vivo HS Human Brain Database (Benchmark): These data from Leon et al.^88^ were captured, processed, and labeled with the previously described method in the process of validating a mixed supervised-unsupervised classification technique. It contains a total of 61 cubes captured from 34 adult patients for the same kinds of tumors as above. The password for this repository can be obtained by contacting the authors.^169^

Later work by Puustinen et al.^154^ attempted to establish a systematic design for a microsurgical hyperspectral database. The architecture of the database was modeled to consider multiple characteristics of captured cubes such as patient information, raw data, red–green–blue (RGB) reconstructions, imaging parameters, manual annotations, pre-operative MRI, regions of interest, calibration standards, and labeled classes. This database is currently access-restricted to their collaborators but is projected to be publicly available in 2024.^154^

Lastly, the Southwest University Longitudinal Imaging Multimodal (SLIM) Brain Database of hyperspectral data has been recently introduced by Martín-Pérez et al.^100^ This dataset contains multimodal data from one line scan hyperspectral camera (Headwall Hyperspec^®^ VIS-NIR E-series, 400 to 1000 nm, 369 effective spectral bands), one snapshot hyperspectral camera (Ximea Corporation MQ022HG-IM-SM5X5-NIR, 665 to 960 nm, 25 spectral bands, 409×217  pixels, 170  frames/s) and an RGB-depth light detection and ranging (LiDAR) (Azure Kinect DK, 3840×2160  pixels, 8-bit depth). The data captured for 193 patients (and counting) at the Hospital Universitario 12 de Octubre in Madrid, Spain, encompasses over a million-pixel spectra labeled semi-automatically by neurosurgeons into five classes: normal (2 subclasses), tumor (10 subclasses), blood (4 subclasses), meninges (2 subclasses), and skull. In addition to raw images, the database contains pre-processed data that remove the effects of depth and noise, hyperspectral cubes cropped to region of interest, generated pseudo-RGB images, and pixel-wise labels. The dataset is available on the database webpage after seeking permission from the authors.^171^ Data from this setup coupled and fused with MRI reconstructions are also available.^172^^,^^173^

#### 3.2.2 Vascular and functional neurosurgery

The data used for hemodynamic imaging in vascular and functional neurosurgery consist of hyperspectral video captured during surgery. One such dataset, captured for imaging interictal epileptiform discharges, exists. This dataset, captured at the Centre Hospitalier de l’Université de Montréal by Laurence et al.,^160^ consists of 8- to 15-m recordings of eight patients aged 24 to 35 treated for epilepsy. Each hyperspectral cube in the video is 256×512  pixels, with 16 spectral channels between 480 and 630 nm. In addition, the data contain intraoperative ECoG recordings from an electrode grid that was manually time-synced with the hyperspectral video, which can be used as the gold standard. These data are available upon request from the authors.^160^

#### 3.2.3 Spinal surgery

Hyperspectral data captured by Ebner et al.^167^ from a bovine calf cadaver in the spinal fusion study described above are available. This dataset was acquired at the Balgrist University Hospital, Zurich, and consists of aligned hyperspectral snapscan (470 to 900 nm, 150 spectral bands, 3650×2048  pixels) and snapshot (665 to 975 nm, 25 spectral bands, 409×217  pixels) cubes. The relevant parts of the hyperspectral cubes were labeled manually by a neurosurgeon. The labels include the various tissue types and implant materials encountered in spinal surgery (skin, fat, muscle, bone, connective tissue, dura, and screws) and are available from the authors upon request.

### 3.3 Visualization Techniques

#### 3.3.1 Neurosurgical oncology

The standard technique for visualizing pixel-wise tissue classification from hyperspectral data is by superimposing a segmentation map (e.g., map of tumor versus normal tissue) over a synthetic RGB (i.e., anatomic) image created from the hyperspectral cube.^113^^,^^115^^,^^131^ However, as classification algorithms that use pixel-wise data do not enforce that neighboring pixels have the same class with high probability (i.e., the classification map is piecewise constant), generating a realistic map requires integrating spatial information. Therefore, several methods from the HELICoiD project^115^^,^^122^^,^^128^^,^^129^^,^^131^^,^^174^ [Figs. 3(a), 4, and 5] use a mixed pixel-wise wide-field approach that makes use of both spatial and spectral information. This approach uses a k-nearest neighbor-based algorithm based on matching and averaging non-local neighborhoods^175^ to combine pixel-wise supervised classification outputs (e.g., with SVM or RF) with locality information from a single-channel representation of the hyperspectral data (generated with spectral dimensionality reduction). This yields a spatio-spectrally inferred pixel-wise classification map. Further, spectral similarity information is incorporated using a majority voting approach^176^ between this spatio-spectral map and a segmentation map generated with k-means clustering. The result of this pipeline is then overlaid upon a synthetic RGB (anatomic) image to yield a visualization that is faithful to both the spectral and spatial properties of the measured hyperspectral data.

Recently, sophisticated methods to visualize, reconstruct, refocus, and project hyperspectral data and segmentation maps have been developed. Augmented reality-based co-projection of HSI-generated RGB data and neural network-based segmentation labels was implemented on a HoloLens AR headset by Huang et al.^177^ and successfully tested in phantom resection procedures. Although the projection quality was excellent, the frame rate was restricted due to an unoptimized software implementation. Other approaches have explored low-level image processing and imaging operations such as hyperspectral image demosaicing to generate synthetic RGB images consistent with the response of the human eye,^178^ hyperspectral image refocusing to tackle depth variation in the surgical field,^179^ and synthetic white balancing to correct for illumination spectrum variability.^180^

“Vascular, functional, and spine neurosurgery” all use digital overlays of the results of their data analysis on an RGB reconstruction of the surgical field.^92^^,^^101^^,^^104^^,^^156^^,^^157^^,^^160^^,^^163^^,^^181^

### 3.4 Clinical Results

Clinical studies using the optical systems and computational methods described above have shown the potential for surgical utility of HSI in reflectance mode for neurosurgery. Here, we review the results from clinical studies performed and present a summary of their statistics and findings in Table 3.

**Table 3:** Clinical validation of hyperspectral imaging systems for neurosurgical applications.

Clinical aim	Target pathologies	Number of acquisitions	Total labeled samples	Imaging setup	Findings	Data
Neurosurgical oncology—reflectance						
Tumor identification, Salvador et al.^112^ and Fabelo et al.^113^	Primary tumors	31 cubes from 22 procedures	19k tumor spectra 104k background 12k normal spectra	Intraoperative (craniotomy)	Pixel-wise hyperspectral data accurately delineates the primary tumor from normal tissue with high sensitivity	No
Tumor detection and type identification, Fabelo et al.^96^	Grade IV glioblastomasLung and renal metastases	13 patients	10k primary tumor spectra 2k metastasis spectra 13k normal spectra	Intraoperative (craniotomy)	Pixel-wise hyperspectral data accurately delineates the primary tumor and metastasis from normal tissue with high sensitivity	No
Tumor identification speedup, Madroñal et al.^97^	Primary tumors	1 patient	19k total spectra	*Ex vivo* imaging	Near real-time SVM classification can be achieved with parallel processing	No
Dimensionality reduction with semantic tumor segmentation, Ravi et al.^114^	Mix primary tumors and metastases	33 cubes from 18 patients	66k tumor spectra 57k normal spectra	Intraoperative (craniotomy)	Fast deep learning–based embedding can effectively reduce dimensionality for semantic segmentation	No
Tumor and blood vessel identification, Fabelo et al.^122^	Grade IV glioblastomas	5 cubes from 5 patients	9k tumor spectra 11k normal spectra 17k blood spectra 8k background	Intraoperative (craniotomy)	Mix spatial–spectral classification with a supervised–unsupervised approach can yield accurate segmentation at surgical frame rates, and a public database can promote further research	Public
Tumor and blood vessel identification and tumor type prediction, Fabelo et al.^115^	Grade III and IV primary tumorsRenal, lung, and breast metastases	36 cubes from 22 patients	14k primary tumor spectra 2k metastasis spectra 117k normal spectra 57k blood vessel spectra 186k background spectra	Intraoperative (craniotomy)	Mix spatial–spectral classification with a supervised–unsupervised approach can yield accurate segmentation at surgical frame rates	No
Tumor identification, Ayaz et al.^136^	Grade IV glioblastomas	26 cubes from 16 patients	11k tumor spectra 102k normal spectra 39k blood vessel spectra 106k background spectra	Intraoperative (craniotomy)	Deep learning techniques have promise in tumor identification and 1D per-pixel DNNs perform comparably with 2D full-field CNNs	No
Hyperspectral band selection, Martinez et al.^99^	Grade IV glioblastomas	26 cubes from 16 patients	11k tumor spectra 102k normal spectra 38k blood vessel spectra 118k background spectra	Intraoperative (craniotomy)	Combinatorial optimization can help select the most informative channels for tumor identification with minimal measurements	No
Tumor and blood vessel identification and tumor type prediction, Fabelo et al.^148^	Grade III and IV primary tumorsRenal, lung, and breast metastases	36 cubes from 22 patients	16k tumor spectra 117k normal spectra 58k blood vessel spectra 186k background (semi-automatically labeled)	Intraoperative (craniotomy)	A robust, labeled database of spectra from various kinds of primary and secondary tumors enables further research where clinical studies cannot be conducted	Public
Tumor and blood vessel identification and phenotype prediction, Martínez-González et al.^119^	Grade IV glioblastomas	13 cubes from 13 patients	124k spectra 602k spectra	Intraoperative (craniotomy) *In vitro* H&E	Hyperspectral imaging can potentially delineate tumor phenotypes in the operating room	No
Brain tissue classification, Cruz-Guerrero et al.^116^	Glioblastoma multiforme	11 cubes from 8 patients	74k spectra	Intraoperative (craniotomy)	Blind linear unmixing-based approaches can speed up hyperspectral tissue classification by 400×	No
Brain tissue classification, Ruiz et al.^98^	Glioblastoma multiforme	4 cubes from 4 patients	6k tumor spectra 11k normal spectra 1.6k venous spectra 600 arterial spectra 4.3k dura spectra (semi-automatically labeled)	Intraoperative (craniotomy)	Hyperspectral imaging shows the potential to segment normal tissue and background into subclasses	No
Testing the surgical feasibility of a hyperspectral imaging workflow, Mühle et al.^87^	N/A	N/A	N/A	Intraoperative (craniotomy)	A surgical microscope-mounted snapshot sensor can be readily integrated into the surgical workflow with minimal disturbance to the staff	N/A
VNIR-NIR data fusion, Leon et al.^147^	Primary tumors	N/S	2.6M spectra	Intraoperative (craniotomy)	Spatial registration methods for parallel VNIR and NIR cameras have the potential to extend VNIR classification features by incorporating NIR information	Public
Tissue component reflectance spectra similarities in VNIR and NIR ranges, Leon et al.^146^	Primary tumors	6 cubes from 4 patients	8k tumor spectra 10k normal spectra 10k blood vessel spectra	Intraoperative (craniotomy)	VNIR and NIR camera spectra have statistically significant differences between normal and tumor tissues in certain wavelength bands	No
Brain tissue classification, Urbanos et al.^150^	High-grade gliomas	13 cubes from 4 patients	15k tumor spectra 28k normal spectra 3.7k venous spectra 1.3k arterial spectra 15k dura spectra (semi-automatically labeled)	Intraoperative (craniotomy)	Various supervised machine learning algorithms (especially RFs) have the potential to accurately predict subclasses of healthy tissue and background	Request
Testing the surgical feasibility of a light-field hyperspectral system in neurosurgery, MacCormac et al.^89^	N/A	1 patient	N/A	Intraoperative (craniotomy)	A surgical microscope-mounted light-field snapshot sensor running at 1 Hz can be readily integrated into the surgical workflow	Public
Testing deep learning and classical machine learning algorithms for low-grade gliomas, Giannantonio et al.^141^	Low-grade gliomas	15 cubes from 5 patients	8671 total tiles—40 × 40 each	Intraoperative (craniotomy)	RFs, radial basis SVMs, and CNNs have the potential to accurately delineate low-grade gliomas from healthy tissue	No
Benchmarking existing algorithms with a new dataset, Leon et al.^88^	Primary tumors and metastases	62 cubes from 34 patients	N/S	Intraoperative (craniotomy)	Previously proposed classification machine learning algorithms have been tested with a new dataset, showing the potential of hyperspectral imaging for real-time decision-making	Public
Tumor identification, Kifle et al.^90^	Primary tumors	364 cubes from 4 patients	N/S	Intraoperative (craniotomy)	Snapshot HSI systems can potentially accurately delineate tumors from healthy tissue for pediatric neurosurgery	No
Low-grade glioma identification, Vandebriel et al.^93^	Low-grade gliomas	5 patients (and counting)	N/S	Intraoperative (craniotomy)	Snapscan HSI systems integrate easily into the surgical workflow and are potentially useful for segmenting low-grade gliomas from healthy tissue	No
Tumor identification and augmented reality visualization, Sancho et al.^152^	Glioblastoma multiforme	5 video sequences from 5 patients	N/S	Intraoperative (craniotomy)	Hyperspectral classification results can be obtained in real time and projected onto a 3D point cloud for tumor visualization	No
Tumor identification and augmented reality visualization, Martín-Pérez et al.^100^^,^^171^	Primary tumors and metastases	193 patients	N/S	Intraoperative (craniotomy)	A joint hyperspectral 3D LiDAR database can facilitate research into augmented reality applications for visualizing tumor delineation	Public
Neurosurgical oncology—fluorescence						
Residual tumor detection via Photofrin, Yang et al.^209^	Primary tumors	6 patients	N/A	Intraoperative (craniotomy)	Multispectral imaging can delineate residual tumor during PDT	No
Tumor identification, Gebhart et al.^107^	Primary tumors	1 patient	N/A	Intraoperative (craniotomy)	Fluorescence and diffuse reflectance spectra can be distinctive between normal and diseased tissues	No
PpIX concentration estimation, Valdés et al.^67^ and Valdés et al.^84^	Glioblastoma multiforme	12 patients	N/A	Intraoperative (craniotomy)	Diffuse reflectances can be used to correct fluorescence spectra for tissue optical properties, enabling absolute PpIX concentration estimation	No
PpIX concentration estimation, Xie et al.^221^	Glioblastoma multiforme	1 specimen from 1 patient	N/A	*Ex vivo* imaging	Spatial regularization can improve detection threshold and PpIX concentration estimate accuracies	No
PpIX concentration estimation, Bravo et al.^219^	Primary tumors	N/S	N/A	Intraoperative (craniotomy)	Hyperspectral data processing improves the PpIX limit of detection and concentration estimate accuracy	Request
PpIX pharmacokinetics in malignant gliomas, Kaneko et al.^202^	Malignant gliomas	201 biopsies from 68 patients	N/A	*Ex vivo* imaging	Fluorescence in malignant gliomas peaks 7 to 8 h after 5-ALA hydrochloride administration	No
PpIX pharmacokinetics in low-grade gliomas, Kaneko et al.^201^	Low-grade gliomas	81 biopsies from 25 patients	N/A	*Ex vivo* imaging	Fluorescence in low-grade gliomas peaks 7 to 8 h after 5-ALA hydrochloride administration	No
Fluorescence component spectra identification, Black et al.^199^	Primary tumors	275 biopsies from 128 patients	2692 spectra	*Ex vivo* imaging	Including autofluorescence and PpIX secondary peak spectra in unmixing increases sensitivity to PpIX concentration and ratio of PpIX peaks may predict tumor grade	No
Tumor type, grade, glioma margins, and IDH mutation prediction, Black et al.^222^	Primary tumors and metastases	891 cubes from 184 patients	100 to 1000 spectra per biopsy	*Ex vivo* imaging	Corrected tumor fluorescence spectra can predict tissue type, tumor margin, WHO grade, and IDH type accurately	No
Joint correction and unmixing of fluorescence spectra, Black et al.^226^	Primary tumors and metastases	891 cubes from 184 patients	555,666 total spectra	*Ex vivo* imaging	Semi-supervised or unsupervised learning can successfully correct for light–tissue interaction and predict absolute PpIX concentrations	No
Fluorescence component spectra identification, Black et al.^223^	Primary tumors and metastases	891 cubes from 184 patients	555,666 total spectra	*Ex vivo* imaging	A Poisson noise model combined with a spectral library of nine fluorophores fits tumor spectra well without overfitting	Spectral library
Vascular neurosurgery						
Cerebral oxygenation mapping, Mori et al.^101^	Ischemic regions	N/S	N/S	Intraoperative (craniotomy)	Hyperspectral imaging is a promising technique for monitoring intraoperative hemodynamics	No
Distinguishing blood and blood vessels, Laurence et al.^157^	Bleeding	9600 cubes from 1 patient	N/A	Intraoperative (craniotomy)	Hyperspectral imaging can be effective in monitoring intraoperative bleeding	No
Diagnosing cerebral hyperperfusion, Iwaki et al.^156^	Cerebral hyperperfusion	29 patients	N/A	Intraoperative (craniotomy)	Hyperspectral imaging can be effective in predicting hyperperfusion	No
Functional neurosurgery						
Imaging seizures intraoperatively, Noordmans et al.^104^	Epileptiform regions	280 cubes (inf.) from 1 patient	N/A	Intraoperative (craniotomy)	Hyperspectral imaging can delineate epileptiform regions by providing oxygenation and blood volume data	No
Imaging neurovascular coupling, Pichette et al.^92^	Epileptogenic focus	480 cubes from 1 patient	N/A	Intraoperative (craniotomy)	Temporal hemodynamics can be measured in real time through a surgical microscope with hyperspectral imaging	No
Metabolic brain mapping, Caredda et al.^163^	N/A	1 patient	N/A	Intraoperative (craniotomy)	Hyperspectral imaging can produce accurate, high-resolution functional maps correlating well with those acquired with electrical stimulation	No
Imaging hemodynamic response to interictal epileptiform discharges, Laurence et al.^160^	Epileptiform regions	8 to 15 min from 12 patients	N/A	Intraoperative (craniotomy)	Hyperspectral imaging can provide accurate optical feedback about interictal epileptiform discharges	Request
Spine surgery						
Positioning feedback and navigation, Manni et al.^59^	N/A	17 volunteers	N/S	*In vivo* imaging	Hyperspectral imaging can be used for markerless feature tracking for positioning guidance and navigation	No
Tissue classification, Ebner et al.^167^	N/A	1 patient	N/S	Intraoperative (craniotomy)	Hyperspectral imaging integrates into spinal surgical workflow seamlessly and provides reliable spectra meeting surgical constraints	Request

N/S, not specified; N/A, not applicable

#### 3.4.1 Neurosurgical oncology

Clinical studies using HSI in reflectance mode for neurosurgical oncology have focused on brain tissue classification during brain tumor resection (16 studies from 2016 to 2024). These studies have implemented classification algorithms, ranging from classical machine learning (RFs, SVMs, and MLPs)^88^^,^^90^^,^^96^^,^^98^^–^^100^^,^^112^^,^^115^^,^^116^^,^^119^^,^^122^^,^^150^^,^^171^ to modern deep learning architectures (CNNs and recurrent NNs)^136^^,^^141^^,^^150^ (Figs. 4 and 5) with the imaging systems described in Table 1. These algorithms have been shown to be highly accurate, sensitive, and specific for identifying tumors. Some algorithms have been optimized to provide results within ∼1  min^97^^,^^119^^,^^152^ (three studies from 2016 to 2023). Accurate segmentation of a large range of primary tumors, including high-grade gliomas to low-grade gliomas, metastases, and healthy tissue types, has been shown using reflectance hyperspectral data. Further, work toward dimensionality reduction and spectral band selection (two studies from 2017 to 2021) has sought to further reduce data processing and acquisition time to enable real-time feedback for the surgeon.^99^^,^^114^ In addition, clinical studies have calculated the objective measures for this separability based on reflectance spectral similarity between the components (2021)^146^ that tested the ease of integration of these methods into the surgical flow (three studies from 2020 to 2023)^87^^,^^89^^,^^93^ and tested the possibility of augmented reality visualization of the hyperspectral outputs (2023).^152^ To facilitate further development with HSI (e.g., novel applications of machine learning algorithms), several of these studies have made their data either publicly available^88^^,^^89^^,^^100^^,^^122^^,^^147^^,^^148^ or available upon request.^150^

#### 3.4.2 Vascular and functional neurosurgery

Clinical studies have explored the application of HSI for imaging of brain hemodynamics, neurovascular coupling, and vascular or functional pathologies using the hyperspectral systems detailed in Table 1. Vascular neurosurgery clinical studies (three studies from 2014 to 2020) have shown HSI can provide accurate estimates of cerebral oxygenation,^101^ the potential for HSI to diagnose brain bleeding,^157^ and estimating hyperperfusion^156^ from hyperspectral data. Using these oxygenation mapping techniques, four studies between 2013 and 2022 demonstrated how intraoperative HSI can be used to detect seizure activity and map functional areas of the brain using principles of neurovascular coupling^160^ and validation with electrocorticography (Fig. 6). One study^160^ has made their data available upon request to facilitate further algorithmic research.^182^

#### 3.4.3 Spine surgery

As a first translational experience using HSI intraoperatively in spine surgery, Ebner et al.^167^ measured full-field spectra of various components in the scene of a patient undergoing spinal fusion (data available upon request). In addition, there has been clinical evidence of HSI-based skin feature tracking as a useful tool for intraoperative navigation in spine surgery.^59^

## 4 Neurosurgical HSI in Fluorescence Mode

Reflectance-based hyperspectral systems provide excellent pixel-wise tissue classification capabilities. However, as observed in previous studies, the reflectance spectra of normal and tumor tissues can be very similar.^146^ Although these similarities can be tolerated in regions of predominantly healthy tissue or bulk tumor, they can be problematic in areas of diffusely infiltrative tumor, which is the case especially in the margins of gliomas,^41^ where residual tumor is likely to lead to tumor recurrence.

In addition, inter-patient and inter-system variability in the reflectance spectra has shown limited generalization of trained models. For instance, mixed-patient pixel-wise data give high classification metrics (∼99% accuracy and sensitivity).^112^^,^^113^ However, these metrics drop to as low as 80% accuracy and 40% sensitivity^131^ when data are divided patient-wise for classification. Such a significant drop in accuracy and sensitivity highlights the current limitations in generalizing these reflectance-based HSI techniques across patients for guiding brain tumor resections.

Fluorescence-guided surgery (FGS) was introduced as a standard of care technique for high-grade gliomas almost 20 years ago and has been shown to be a safe and effective surgical adjunct to delineate tumor tissue intraoperatively.^9^^,^^183^ FGS “extends” the surgeon’s vision by increasing the contrast between healthy and tumor tissues.^5^^,^^45^^,^^184^^–^^186^ Clinically approved fluorophores for FGS include 5-aminolevulinic acid (ALA)-induced PpIX,^187^^–^^189^ fluorescein sodium (FS),^190^^,^^191^ and ICG^192^^,^^193^ (Fig. 2). These fluorophores selectively accumulate in tumor tissue through various cellular mechanisms^194^ and fluoresce when illuminated with excitation light having an appropriate wavelength. PpIX and FS are typically excited with violet and blue light at 405 and 494 nm, respectively, and fluoresce in the VIS spectrum with emission maxima at 635 nm^188^ and 520 nm,^190^ respectively.^195^ ICG is excited at ∼780  nm and fluoresces with its peak in the NIR at 815 nm.^193^ However, it has been shown to produce significant fluorescence contrast beyond 1000 nm, allowing for imaging in the SWIR range.^196^

**Fig. 7:**
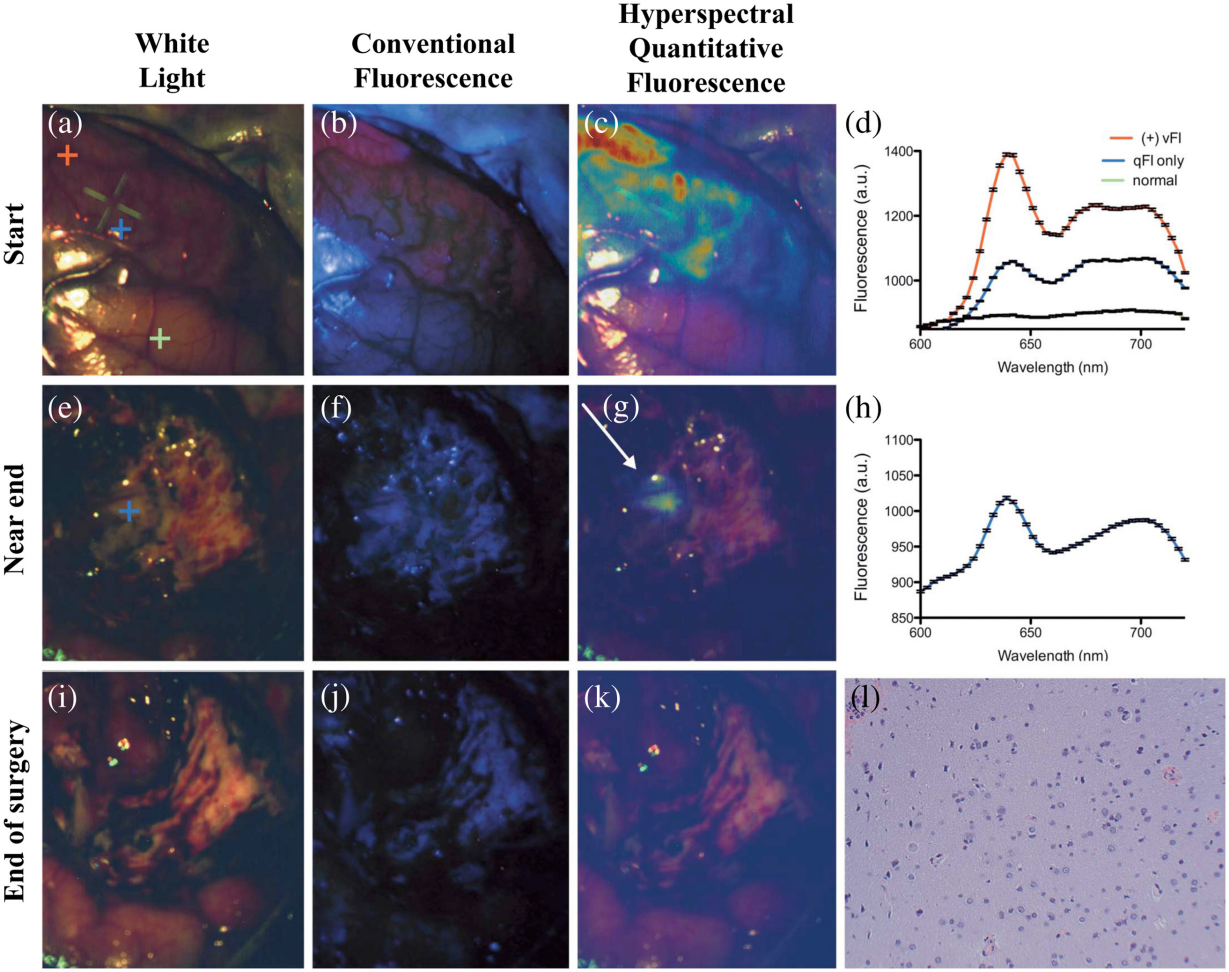
*In vivo* hyperspectral fluorescence imaging of PpIX in a glioblastoma patient. Intraoperative images using a spectral scanning system [Fig. 4(b)] were captured during the resection of glioblastoma with images at the beginning (a)–(c), near end (e)–(g), and end of the surgery (i)–(k). The first three columns show (from left to right) RGB images reconstructed from the hyperspectral cube (white light), co-registered fluorescence images using the conventional fluorescence surgical microscope (conventional fluorescence), and PpIX concentration maps estimated from hyperspectral cubes (hyperspectral quantitative fluorescence). (d) *In vivo* fluorescence spectra acquired from three locations and marked by different colored crosses (+) in panel (a) with a high-intensity PpIX spectrum, and peak in red (+) matches the visible pink fluorescence in the center of tumor (b); an intermediate intensity PpIX spectrum and peak in blue (+) with no visible pink fluorescence is close to tumor in panel (b); and no PpIX spectrum and peak in green (+) matching no visible pink fluorescence far from tumor in panel (b). (h) *In vivo* fluorescence spectra acquired from one location and marked by a blue colored cross (+) in panel (e) show an intermediate intensity PpIX spectrum and peak in blue (+), no visible pink fluorescence in panel (f), high estimated PpIX concentrations in panel (g), and are validated with pathology as tumor-infiltrated tissue in panel (l). In panels (d) and (h), the y-axis shows the intensity of fluorescence emission in arbitrary units, and the x-axis shows the wavelength λ in nanometers. vFI, visible fluorescence with the conventional microscope; qFI, quantitative fluorescence imaging estimates of PpIX. Reproduced from Valdés et al.,^67^ under CC-NC-SA 3.0.

5-ALA-induced PpIX fluorescence has been extensively studied,^197^^,^^198^ validated,^45^ characterized,^199^^–^^202^ and established as a standard in surgery.^195^^,^^203^ PpIX is an intermediate in the hemoglobin synthesis pathway. The mechanisms of PpIX accumulation in tumor tissue are multifactorial (e.g., increased tumor metabolism, tumor proliferation, enzymatic or cellular transporter modifications, and blood–brain barrier breakdown^204^). Studies have clearly demonstrated its utility in guiding resections with excellent diagnostic metrics for tumor tissue identification. PpIX accumulates in tumors to produce significant fluorescence after an oral dose of its precursor, 5-ALA (20  mg/kg)^205^ 2 to 3 h before surgery. Further, PpIX has its largest excitation maximum at 405 nm,^188^ with a broad (>200  nm) Stokes shift between the 405-nm excitation maxima and its emission maximum at 635 nm.^188^ This large Stokes shift allows for effective filtering of excitation light without loss of fluorescence emissions. Further, most of its fluorescence spectrum lies in the domain where tissue scatters light with low hemoglobin absorption and low autofluorescence.^200^ Thus, HSI has been used to isolate PpIX fluorescence from autofluorescence, other fluorescent markers and noise via spectral fitting, and correction for attenuation due to tissue optical properties. The use of spectral-based processing capable with HSI has enabled the detection of “invisible tumors” due to the ability to measure lower levels of PpIX below the visible threshold of conventional clinical systems^67^^,^^199^ (Fig. 7). This increase in sensitivity and preservation of specificity for PpIX fluorescence has been quantified systematically.^206^ We will next discuss HSI systems that leverage these advantages along with associated computational methods.

### 4.1 Imaging Hardware and Software

The first demonstration of multispectral fluorescence imaging in neurosurgical oncology was in 2003 using a wide-field five-band (bandpass spectral filters from Omega Optical^207^ at 495-, 543-, 600-, 640-, and 720-nm center wavelengths; 20-nm bandwidth, 755×484 DVC CCD detector^208^) multispectral system. Here, the authors imaged a fluorescent tumor after exogenous administration of the fluorescent agent, Photofrin,^209^ with a total acquisition time of 15 s. This study concluded that multispectral imaging has the capability to separate Photofrin fluorescence from a background with a 10:1 signal-to-background ratio. Further, it hypothesized that multispectral data could estimate Photofrin concentrations, with a detection limit of 50 to 100  ng/ml at 0.5-mm depth inside tissue-mimicking phantoms. However, this work assumed that tissue is homogeneous, causing these estimates to be accurate only when tissue optical properties matched the validation phantoms.

As noted before, the first hyperspectral fluorescence imaging was in 2007, where Gebhart et al.^107^ developed an HSI system that consisted of a Varispec VIS-20 LCTF from Cambridge Research Instruments, Inc.^108^ coupled with a 512×512 PhotonMax EMCCD camera^109^ mounted on a surgical microscope to measure intraoperative autofluorescence and diffuse reflectance spectra in one patient. The authors found that a value less than 1.25 for the ratio of autofluorescence at 460 nm to diffuse reflectance at 700 nm was highly diagnostic for tumor tissue.

Valdés et al.^67^ developed a similar hyperspectral system and implemented the first intraoperative approach to correct fluorescence signals for the distorting and attenuating effects of tissue optical properties in 12 patients with brain tumors [Fig. 3(c)]. They imaged the diffuse reflectance at excitation and emission wavelengths and fluorescence, followed by implementation of a correction algorithm^67^^,^^210^^,^^211^ (i.e., a spectrally constrained dual-band normalization algorithm) for use in 5-ALA-PpIX FGS. Similar to the work by Gebhart et al.,^107^ this approach used a Varispec LCTF coupled to a pco.pixelfly camera and custom optical adapter^110^ unto a surgical microscope modified for fluorescence imaging (Zeiss OPMI Pentero). The surgical field was imaged under white light and 405-nm illumination respectively^67^^,^^84^^,^^211^ to measure fluorescence spectra and reflectance with a total maximum acquisition time of <16  s. The measured fluorescence spectrum $F_{\text{raw}}(\lambda )$ was corrected by an empirical factor inversely proportional to the excitation reflectance $R_{\mathrm{exc}}$ and power law proportional to the emission reflectance $R_{\mathrm{em}}$.

$$F_{\mathrm{corr}}(\lambda )=\Omega \frac{F_{\text{raw}}(\lambda )}{R_{\mathrm{exc}}R_{\mathrm{em}}^{-0.7}}.$$

The corrected fluorescence spectrum was fit to a weighted sum of basis spectra for fluorophores of interest (e.g., PpIX, fluorescein sodium, and tissue autofluorescence) to isolate only PpIX or FS fluorescence. Thus, the estimated corrected PpIX values were found to be directly proportional to absolute PpIX concentrations. This correction allowed the detection of PpIX concentrations as low as 20  ng/ml, which was significantly lower than the lowest concentrations of 600 to 1000  ng/ml found in visually fluorescent (i.e., red-pink visual fluorescence through surgical oculars) high-grade glioma tissues. Further, these results were encouraging as they indicate the ability to detect low yet diagnostically significant PpIX concentrations to identify low-grade glioma and infiltrative margins that are usually “invisible” with conventional techniques^49^^,^^51^^,^^67^^,^^189^^,^^212^^–^^214^ (Fig. 7). This work concluded that a threshold of 100  ng/ml had a positive predictive power of >90% for tumor tissues. The HSI approach by Valdés et al.^67^ was further validated in additional studies demonstrating improved detection capabilities in clinical ALA-PpIX FGS.^84^ In further work by Valdés et al.,^211^ a more sensitive pco.edge camera^215^ allowed lower acquisition times of 1 to 2 s with the same detection limit. An even more sensitive EMCCD camera^216^ from Nüvü cooled to −85°C further decreased the limit of detection to 1  ng/ml, comparable to point spectroscopy methods^217^ at a maximum total acquisition time of 5 s. This correction method was further applied to pediatric brain tumors, where the limit of visual detection was determined to be 200  ng/ml,^218^ and the lower limit of detection for PpIX was 20  ng/ml. These were all validated with tissue-mimicking phantoms consisting of a solution of PpIX mixed with an absorber (e.g., hemoglobin and yellow food dye) and a scatterer (e.g., intralipid emulsion).^67^ Known fluorophore concentrations in these phantoms can be used to map the corrected fluorescence to absolute PpIX concentrations and evaluated for accuracy metrics such as linearity (i.e., $R^{2}$ value and mean percentage errors).

Spectrally constrained dual-band normalization has been systematically evaluated for its accuracy in correcting the raw fluorescence signal for tissue optical properties, its highly sensitive estimates of fluorophore concentrations (i.e., PpIX),^67^^,^^210^^,^^211^^,^^219^^,^^220^ its reproducibility by different clinical and research teams and HSI systems,^94^^,^^221^^–^^223^ and its diagnostic utility with greater sensitivity, negative predictive values, and overall accuracy for tumor detection compared with visual expert evaluation. Specifically, Lehtonen et al.^206^ found that visual assessments yielded 63% accuracy, 48% sensitivity, 92% specificity, and 340  ng/ml minimum limit of detection for PpIX. Meanwhile, an HSI system based on a standalone Senop HSC-2 camera (500 to 900 nm, up to 1000 spectral bands, 1024×1024  pixels, 150  frames/s)^224^ yielded 96% accuracy, 100% sensitivity, and 86% specificity, and 16  ng/ml minimum limit of detection (16 samples with PpIX and eight control samples; number of patients not specified).

**Fig. 8:**
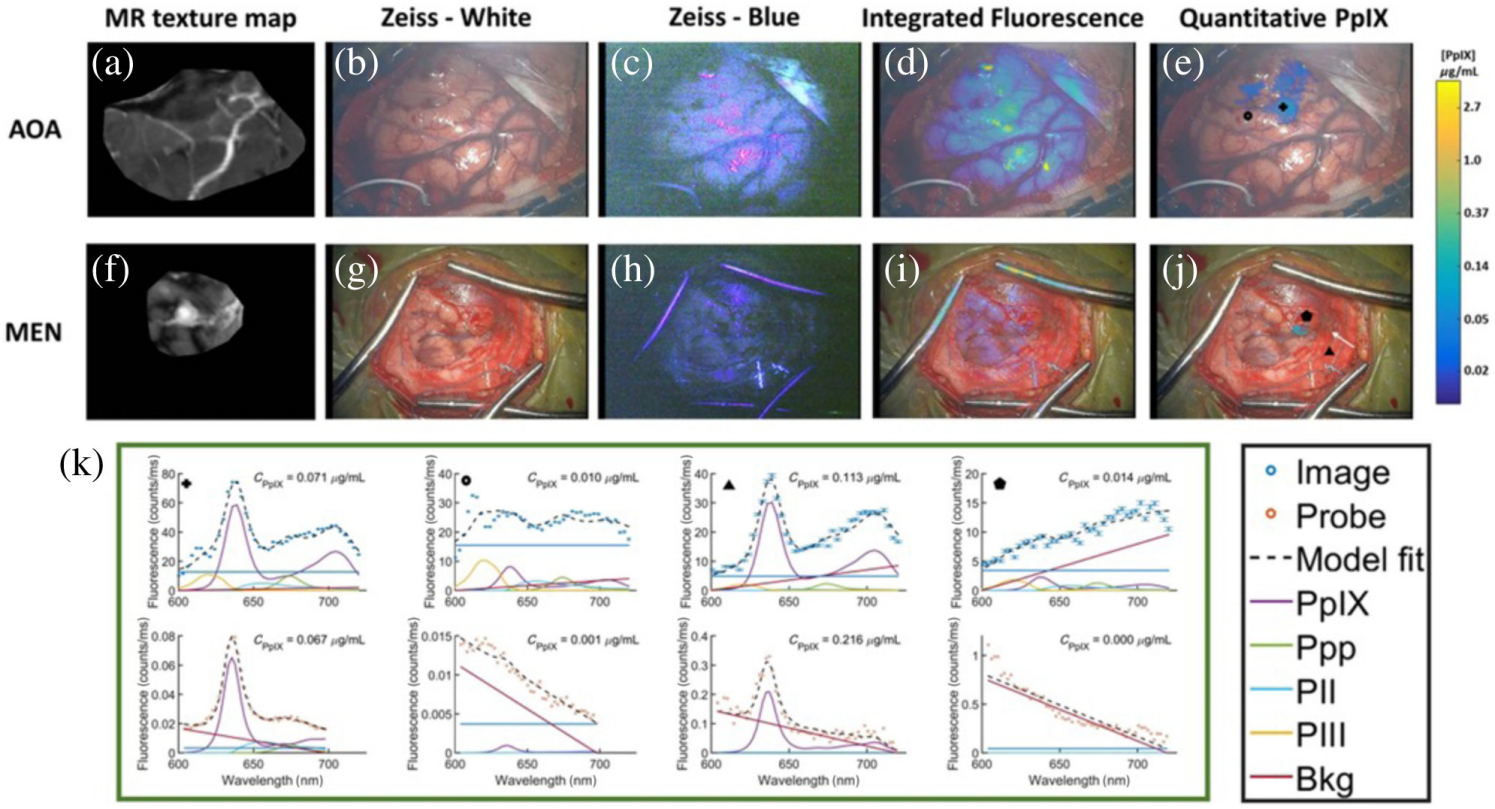
Comparison of HSI to point spectroscopy. Point spectroscopy provides gold standard spectrally resolved measurements and PpIX concentration estimates that can be used to validate the estimates from hyperspectral processing. HSI extends the applicability of fluorescence guidance to WHO grade III anaplastic oligoastrocytomas (AOA) (a)–(e) and meningiomas (MEN) (f)–(j), where the PpIX concentration is significantly less than the limit for visual fluorescence. (k) Fluorescence spectra fit and estimated PpIX concentrations from HSI (top) and point spectroscopy measurements (bottom). MR texture map = matching MRI 2D image; Zeiss—white = white-light image from a conventional Zeiss microscope; Zeiss—blue = fluorescence image from a conventional Zeiss microscope; integrated fluorescence = map of fluorescence calculated from the area under the fluorescence spectrum from 620 to 650 nm; quantitative PpIX = map of PpIX concentration estimates. Reproduced from Bravo et al.,^219^ under CC-BY 4.0.

Bravo et al.^219^ have shown in three patients that corrected concentration estimates (with spectral fitting to isolate PpIX) correlate strongly with point spectroscopy estimates^220^ (linear fit r=0.98) when compared with uncorrected estimates (linear fit r=0.91 accounting for other fluorophores, linear fit r=0.82 not accounting for other fluorophores) (Fig. 8).

Xie et al.^221^ developed a Bayesian reconstruction method based on spatial regularization and tested it on one tissue specimen from a glioblastoma patient. This approach defines reconstruction in terms of a total variation-regularized minimization problem

C^(x,y)=arg minC(x,y)[∑x,y,λ(Fraw(x,y,λ)−Ω(1−Rexc(x,y,λ))Rem2.6(x,y,λ)C(x,y))2+Γ‖∇C(x,y)‖1].

The first term here, based on previous point spectroscopy analysis,^85^ attempts to make the reconstruction of C(x,y) faithful to the measurement of $F_{\text{raw}}(\lambda )$. Here, Ω is a factor that maps corrected fluorescence intensity to concentration, and Γ is a regularization factor that decides the smoothness of the reconstruction. This reconstruction lowers the detection limit to 10  ng/ml using an uncooled ORCA-Flash4.0 EMCCD sensor from Hamamatsu Photonics with 26 s of total acquisition and processing time. Such low detection levels would be particularly useful for detecting low, but diagnostically significant PpIX levels in low-grade gliomas.^220^ Further computational work used an unspecified sCMOS camera^225^ with the Sony IMX252 sensor by Black et al.^199^ and a pco.edge camera (14  ng/ml minimum detection limit).^94^^,^^219^^,^^226^

Finally, Black et al.^222^ used machine learning–based approaches on the unmixed fluorophore contributions to predict the following tumor properties in 891 hyperspectral measurements from 184 patients with multiple brain tumor histology types: tumor type (12 categories, test accuracy 85%), tumor margin location (tumor bulk, infiltrative margin, and healthy tissue altered due to tumor, test accuracy 96%), isocitrate dehydrogenase enzyme (IDH) gene mutation type (mutated and normal, test accuracy 86%), and tumor grade (II–IV, test accuracy 93%). In addition, PCA variation analysis revealed that the five fluorophores mentioned above were the most likely components explaining the dataset spectra under the assumption of Gaussian noise.^222^ Incorporating the more physically accurate Poisson unmixing model, with a dataset containing 555,666 spectra, allowed Black et al.^223^ to unmix fluorophores previously impossible due to their small proportion and thus building up a “spectral library” containing ${\mathrm{PpIX}}_{620}$ (see next paragraph), ${\mathrm{PpIX}}_{634}$, reduced nicotinamide-adenine dinucleotide (NADH), flavin adenine dinucleotide (FAD), flavins, lipofuscin, melanin, elastin, and collagen as its members. Finally, deep learning–based architectures have incorporated the non-linear wavelength-dependent effects not taken into account by the previous algorithms to correct and unmix fluorescence spectra with a semi-supervised architecture.^226^ This approach yielded more realistic and smooth estimates of PpIX concentration maps as well as removing imaging artifacts such as specularities.

As mentioned above, correction methods, such as spectrally constrained dual-band normalization, commonly undergo validation using fluorescent tissue-mimicking liquid phantoms. However, a recent study by Suero Molina et al.^227^ has proposed a photostate of PpIX that contributes a fluorescence spectrum shifted to a peak at 620 nm that likely occurs naturally in tissue, but not in such phantoms. The presence of this photostate (called ${\mathrm{PpIX}}_{620}$ as opposed to the usual ${\mathrm{PpIX}}_{634}$) impacts the accuracy of conventional linear fitting models which use the basis spectra of ${\mathrm{PpIX}}_{634}$, PpIX photoproducts and autofluorescence from NADH, lipofuscin, and flavins. Therefore, incorporation of the ${\mathrm{PpIX}}_{620}$ spectrum into linear fitting models has been proposed to improve the accuracy of the spectral fit in dimly fluorescent areas (e.g., low-grade gliomas and infiltrative regions of high-grade gliomas). This also lowers false positives by removing the spurious contribution of ${\mathrm{PpIX}}_{620}$, yielding the true ${\mathrm{PpIX}}_{634}$ spectrum and therefore accurate, lower ${\mathrm{PpIX}}_{634}$ estimates.^199^ Further, additional studies have noted the proportion of the two photostates (i.e., the overall blue shift of the PpIX spectrum) correlates with tumor grades of tissues.^214^

This LCTF design provided a small footprint to enable HSI with high spatial resolution at user-defined spectral resolutions and acquisition times in the order of seconds. Although this HSI design and subsequent implementations have been translated into the operating room given their integration with commercial surgical microscopes, they suffer from one major limitation for widespread surgical use: image acquisition from these systems requires spectral scanning (i.e., an image for every wavelength of interest with a finite amount of camera exposure for each wavelength to reconstruct a full hyperspectral cube). As such, these HSI systems have limited intraoperative utility for widespread use because they do not provide real-time surgical guidance. To address this limitation, a recent snapshot HSI system that used a series of birefringent crystals was developed by Marois et al.^228^ to capture 64 spectral channels at a time. This system achieved a frame rate of 4 to 6  frames/s over a broad wavelength range (425 to 825 nm, 64 spectral bands, 600×400  pixels) and subsequently implemented a spectrally constrained dual-band normalization technique as well.

### 4.2 Clinical Results

Clinical studies using HSI in FGS have focused mostly on tissue classification for improving tumor detection (Table 3). The first study sought to detect residual tumors with a limited number of (multispectral) images^209^ coupled to visual inspection of these channels. The first quantitative clinical studies, carried out by Valdés et al.,^67^ performed unmixing of fluorescent components of tissue via fluorescence spectrum fitting and correction of PpIX fluorescence intensity for attenuation due to light-tissue interaction to estimate absolute pixel-wise tissue concentrations^67^^,^^84^ on 12 patients undergoing brain tumor resection (Fig. 7). Subsequent work from this group showed improvements in accuracy and sensitivity for PpIX detection.^219^ These corrections were further incorporated into a spatially regularized optimization for smooth and accurate estimates of PpIX concentration maps.^221^^,^^226^ Further, the autofluorescence properties of tissue were characterized in two studies to incorporate them into the unmixing algorithms, using an increasing number of components and known compounds (e.g., PpIX photoproducts and differing PpIX states)—one analyzing 2692 *in vivo* spectra from 128 patients^199^ and one building a spectral endmember library from 555,666 fluorescence spectra measured from 891 *ex vivo* sample measurements.^223^ The coefficients of the resulting fluorescence spectrum fit were shown to be useful for predicting properties of tumors such as type, margin, grade, and IDH mutation status^222^ in 891 spectra from 184 patients. Further, to optimize the dose and administration time of 5-ALA, hyperspectral studies were performed to estimate the pharmaco-kinetics of PpIX inside tissue—one with 81 spectra from 25 patients for low-grade gliomas^201^ and one with 201 spectra from 68 patients for malignant gliomas.^202^ These studies showed an optimal post-dose time of 7 to 8 h at which PpIX tumor fluorescence signal peaks.

The results of these studies point toward the potential for HSI to enhance fluorescence feedback to serve as an improved surgical adjunct. One of these HSI studies has made its dataset available upon request,^219^ whereas another offers the spectral library constructed during its analysis^223^ to facilitate further research. Exact PpIX concentrations, which are determined by correcting its fluorescence spectrum from the distorting effects of tissue optical properties and unmixed from autofluorescent and other fluorescent components in tissue, can increase the accuracy of predicting tumor presence, whereas the unmixed autofluorescent parts predict tumor properties with machine learning. This, combined with the optical functional and vasculature mapping from the previous section, will allow for all-optical joint visualization of anatomy and tumor for safe and accurate tumor resection.

## 5 Future Perspectives

As discussed in the previous sections, there is substantial evidence supporting the potential of HSI for intraoperative visual feedback. HSI systems, particularly those utilizing snapshot and snapscan techniques, are convenient for clinical deployment due to their small footprint and near-real-time acquisition capabilities. Co-developed computational methods have demonstrated excellent results in the accurate delineation of tumor pathology and normal tissue. These systems have also enabled non-invasive ECoG-style brain mapping of metabolically active tissue to visualize functional connectivity and hemodynamic inference of molecular metabolic parameters such as oxyhemoglobin, oxCCO concentrations, and oxygen saturation. Prototype augmented reality systems have shown promise in accurately projecting hyperspectral results onto the surgical field in 3D. Integrating these capabilities together can create a powerful, unified, non-invasive, optical 3D visualization system that seamlessly integrates into the existing surgical hardware and workflow. Such a system will provide the surgeon with information far richer than can be done with traditional visual methods or with an RGB camera displayed on 2D monitors.

However, there are areas that need improvement to enhance these guidance techniques. The most critical aspect is the framerate of the final hyperspectral outputs. The pipeline leading to these outputs involves acquisition, processing, and projection, each of which needs optimization. By individually or jointly refining these components, the final framerate can be brought closer to real-time, significantly improving the system’s utility in surgical settings.

Among the variety of HSI implementations discussed in Sec. 2.2, snapshot and snapscan hyperspectral systems^70^^,^^71^^,^^92^ coupled with a surgical microscope seem to be the most practical for immediate clinical translation. Even with these solutions, more work needs to be done to increase the spatial resolution of the hyperspectral cube. One possible approach in this direction is upsampling the low-spatial-resolution hyperspectral cube with bilateral upsampling^229^ and pansharpening^230^ algorithms. To make the more commonly used line-scan hyperspectral imaging systems practical for surgical guidance, their quantum efficiency needs to be increased and noise floor needs to be decreased—both of which can be achieved using cooled emCCD cameras,^216^ among other systems.

Another potential direction of acquisition speedup is dimensionality reduction. Because hyperspectral channels have certain spatial regularity (nearby pixels of nearby channels have close intensity values with high probability), not all the entries in the hyperspectral cube are fully independent. Therefore, it is possible to measure subsets of the hyperspectral cube, or an approximation to it, while still extracting the required information. Examples of this approach are selecting specific, most important spectral channels;^90^^,^^99^^,^^118^^,^^145^^,^^147^ implementing pre-calculated programmable spectral filters matched with the combination of tissue components needed;^78^ and measuring low-rank approximations to the hyperspectral cube.^76^ Even with these existing methods, selecting the free parameters—number of channels to use, filter shapes, and rank of the approximation—remains an open problem, requiring an analysis of the statistics of the hyperspectral data and the propagation and noise model of the imaging system.^160^ However, a balance needs to be achieved among speed (e.g., real-time imaging), quality of HSI data (e.g., high spatial resolution, high spectral resolution, and high signal to noise), and/or cost (e.g., light-field technologies) that would be of clinical value. HSI is still in its infancy as an intraoperative imaging modality, and as newer systems are translated into clinical use for specific applications (e.g., HSI for FGS of gliomas), the right balance among speed, data quality to provide clinical value, and costs will likely determine the impact HSI will have as an intraoperative imaging modality.

Current computational algorithms and their implementations need significant work to bring them up to the required speeds. Condensed data input from imaging systems as described above, combined with parallel computational implementations of optimized algorithms on platforms such as field-programmable gate arrays,^125^ can allow for this acceleration. Improved classification algorithms, optimized for sensitivity to the pathology under consideration and modified to use the condensed data above, can lessen the required computational load. The ability to process hyperspectral images fast would imply that it is possible to also process hyperspectral videos, opening up avenues for applying previously developed computer vision techniques for instrument and feature tracking, manipulation, and guidance. To incorporate these results into a comfortable 3D display equipped for surgery or telesurgery, optimized implementations of augmented reality projection methods prototyped in the literature^177^ need to be developed. Lastly, to jointly optimize all the components above, methods to simulate the entire pipeline—emission at the light source, propagation through the scene and image formation at the camera—must be developed to ease the requirement of prototyping the corresponding HSI systems.^162^^,^^165^

Due to the narrow focus of existing clinical studies on certain kinds of pathologies, each clinical study suffers from a low number of patients.^113^^,^^115^^,^^141^^,^^221^^,^^231^ The need for larger and ultimately randomized controlled clinical studies—in terms of pathologies and imaged tissue properties^90^^,^^96^^,^^119^^,^^145^^,^^150^^,^^151^^,^^222^^,^^223^ and demographics^156^^,^^160^ is an essential step forward in establishing hyperspectral imaging as a standard in intraoperative guidance. Further, clinical HSI studies have not reported on non-randomized patient outcomes (e.g., overall survival, progression free survival, and rates of seizure freedom). In addition, it is vital to standardize the protocol of such clinical studies so that results are reproducible and comparable across studies,^160^^,^^231^ to standardize data formats and schematics so that they can be parsed and re-utilized easily, and to set specific goals to be achieved with each clinical approach.^89^ These studies must include in them an analysis of inter-patient data and statistics variabilities^125^ and methods to counter them to ensure consistent results across time. In addition, it is necessary for clinical studies to also consider the ease and complexity of use of the studied system and to note the experience of the operating room (OR) team post-study for further refinement.^89^^,^^167^

As a result of the relatively few clinical studies and privacy concerns, as noted in previous work,^90^^,^^99^^,^^113^^,^^115^^,^^141^^,^^152^^,^^222^^,^^231^^,^^232^ there is a lack of publicly labeled hyperspectral data to enable the development of computational techniques at venues of high expertise in artificial intelligence, where clinical studies cannot be conducted. This is especially the case with rare tumors and vascular and functional disorders. The available datasets are all semi-automatically labeled with input from a neurosurgeon or a pathologist, which has the possibility of human error. Therefore, there is a need for fusing HSI with other, more established imaging modalities, such as MRI, for automatic labeling of hyperspectral images.^172^^,^^173^ In addition, in infiltrative tumors, where it is impossible to draw a sharp boundary between tumor and healthy tissues, a method for fuzzy margins is needed to perform accurate labeling,^222^ which can be achieved with co-registered MRI data and MRI classification algorithms. Fusion with MRI also allows for estimation of brain shift and joint intraoperative feedback from both modalities.^59^

Furthermore, all the HSI systems described here image light in the visible, NIR, and SWIR ranges of the electromagnetic spectrum. Light in this range has limited penetration depth. Therefore, these HSI systems have limited ability for imaging deep in tissues,^233^ typically lesser than a centimeter of depth. Meanwhile, techniques such as MRI, US, and intraoperative neuronavigation provide 3D information deeper inside brain tissue. A fusion of these techniques will allow the surgeon to interpret these sources of complementary information—*in vivo* surface/subsurface molecular information from HSI, *in vivo* subcentimeter structural information from US, 3D structural information at one time point during surgery from intraoperative MRI, and correspondences with 3D pre-operative information with intraoperative neuronavigation.^46^

The widespread adoption of intraoperative HSI depends on the success of the aspects of future work listed above and the practicality of the resulting optimized methods. The success of these developed HSI methods in pre-clinical work and clinical studies opens up possibilities for commercial miniaturization, cost reduction, and integration into existing surgical microscopes and visualization software and hardware and will drive further research with large-scale funded projects such as the HELICoiD.^113^ If effective enough, techniques developed in neurosurgical HSI can be applied to minimally invasive procedures, procedures in other surgical subspecialties, and data generation for education and surgical training tools. In summary, supported by modern techniques from imaging, computation, and visualization, and driven by clinical interest, hyperspectral imaging has the potential to be a clinical standard of care in neurosurgery.

## Data Availability

This review paper was based on a literature survey of hyperspectral imaging in neurosurgery performed using standard tools such as Google Scholar and PubMed. Therefore, there is no code or data accompanying this paper. All the claims, results, and data we quoted in the paper are accompanied by citations to their original research publications.
